# [60]Fullerene–Cluster Hybrids as Boron‐Dense Delivery Nanoplatforms

**DOI:** 10.1002/smsc.70413

**Published:** 2026-10-07

**Authors:** Jennifer Patino‐Alonso, Mónica Muñoz‐Úbeda, Iván López‐Montero, M. José Ruiz‐Magaña, Carmen Ruiz‐Ruiz, Ignacio Porras, Miquel Nuez‐Martínez, Rosario Núñez, Beatriz M. Illescas, Justo Cabrera‐González, Nazario Martín

**Affiliations:** ^1^ Departamento de Química Orgánica Facultad de Química Universidad Complutense Madrid Spain; ^2^ Departamento de Química Física Facultad de Química Universidad Complutense Madrid Spain; ^3^ Instituto de Investigación Sanitaria Hospital Doce de Octubre (imas12) Madrid Spain; ^4^ Instituto Pluridisciplinar Universidad Complutense Madrid Spain; ^5^ Instituto de Biopatología y Medicina Regenerativa Centro de Investigación Biomédica Universidad de Granada Granada Spain; ^6^ Departamento de Física Atómica, Molecular y Nuclear Facultad de Ciencias Universidad de Granada Granada Spain; ^7^ Institut de Ciència de Materials de Barcelona (ICMAB‐CSIC) Cerdanyola del Vallès Spain; ^8^ Departamento de Química en Ciencias Farmacéuticas Facultad de Farmacia Universidad Complutense Madrid Spain; ^9^ IMDEA‐Nanoscience Madrid Spain

**Keywords:** BNCT, boron clusters, carbon nanostructures, fullerenes, molecular materials, nanomedicine

## Abstract

Boron Neutron Capture Therapy (BNCT) relies on the accumulation of boron‐10 within tumor cells to induce highly localized cytotoxicity upon neutron irradiation. The development of boron delivery systems with high boron content, biocompatibility, and efficient cellular uptake remains a critical challenge. Herein, we report the design and synthesis of unprecedented fullerene–boron cluster hybrids that integrate the multivalent scaffold of [60]fullerene with anionic boron clusters. Two [60]fullerene hexakis‐adducts bearing twelve peripheral cobaltabisdicarbollide (COSAN) or *closo*‐dodecaborate boron clusters have been prepared through a Cu(I)‐catalyzed azide–alkyne cycloaddition strategy. The resulting nanostructures incorporate up to 216 boron atoms while preserving the structural integrity of both the fullerene core and the anionic boron clusters. Comprehensive spectroscopic and electrochemical characterization confirms the successful formation of the hybrids. To enable biological evaluation, the compounds are formulated into liposomes, showing excellent cytocompatibility and efficient cellular internalization in mouse embryonic fibroblasts and Cal33 human head and neck cancer cells. Confocal microscopy reveals predominant endolysosomal localization without significant cytotoxicity at therapeutically relevant boron concentrations. These results establish fullerene–boron cluster conjugates as a new class of boron‐rich nanomaterials and highlight their potential as boron delivery agents for future BNCT studies.

## Graphical abstract

[60]Fullerene‐based nanoplatforms functionalized with boron clusters enable the delivery of high boron payloads into cells. These structures exhibit good biocompatibility and efficient cellular uptake, accumulating in endolysosomal compartments. Their high boron density makes them promising candidates as boron delivery agents.

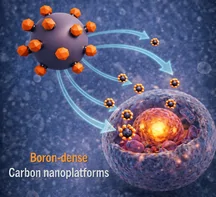

## 1 Introduction

The development of nanomaterials for cancer therapy increasingly seeks to integrate molecular components that combine biocompatibility, chemical tunability, and therapeutic efficacy [1, 2]. Within these nanometric materials, carbon‐based nanostructures have gained relevance in the biomedical field, where their unique physicochemical properties have been exploited for applications ranging from drug delivery and imaging to photothermal and photodynamic therapies [3].

Among them, [60]fullerene is distinguished by its exceptional structural, electronic, and physicochemical properties [4]. This highly symmetrical carbon nanostructure can be selectively functionalized through diverse approaches, allowing the controlled presentation of multiple ligands in a well‐defined 3D geometry [5, 7]. In particular, the preparation of [60]fullerene hexakis‐adducts by the Bingel–Hirsch cyclopropanation reaction has led to globular nanocarbon scaffolds with octahedral geometry [8]. This strategy has reported biocompatible C_60_ hexakis‐adducts derivatized with biomolecules such as carbohydrates, peptides, or amino acids, reinforcing the potential of [60]fullerene as biocompatible nanoscaffold for biomedical applications [9, 10, 11].

In this context, biocompatible hybrids such as glycofullerenes have demonstrated remarkable efficacy in blocking the carbohydrate‐recognition domains of receptors targeted by emerging viruses, including Zika, Dengue, and Ebola [12, 15]. Particularly, striking results were reported by our group for nanoassemblies constructed from a central C_60_ fullerene scaffold decorated with 12 glycofullerenes—also referred to as tridecafullerenes—collectively presenting 360 carbohydrate moieties. These architectures achieved potent inhibition of Ebola‐pseudotyped particles infection, with IC_50_ values in the picomolar range [16]. More recently, we have published results where glycofullerene‐porphyrin conjugates are able to inhibit up to 90% of SARS‐CoV‐2 trans‐infection [17]. This versatility to modulate glycan density has proven effective in the design of C_60_‐based glycoconjugates as viral entry inhibitors [18, 19].

Fullerene‐based nanomaterials exhibit valuable photophysical properties that enable their use as photosensitizers, generating reactive oxygen species upon light irradiation. This has supported their application in photodynamic strategies, including anticancer and antimicrobial therapies [20, 21, 22]. The biocompatibility of modified C_60_, together with its high capacity for surface modification, make it an excellent scaffold for the transport and delivery of therapeutic drugs [23, 24], a potential further strengthened by the enhanced permeability and retention (EPR) effect of nanostructures in tumor tissues [25, 26].

On the other hand, Boron Neutron Capture Therapy (BNCT) has reemerged as a binary cancer treatment that depends on the accumulation of ^10^B within tumor tissues, followed by irradiation with low‐energy neutrons [27, 28]. This process generates highly cytotoxic α‐particles and ^7^Li nuclei directly inside cancer cells, resulting in localized cell death with minimal impact on surrounding healthy tissue. A major challenge for effective BNCT has been the development of boron delivery agents. Although boronophenylalanine (BPA) and sodium borocaptate (BSH; sodium mercaptoundecahydrododecaborate) represent important milestones in the clinical translation of BNCT, their application is constrained by several limitations, including a relatively low boron payload per molecule, limited tumor selectivity, and the need for efficient accumulation within tumor tissue [29]. Recent advances in nanotechnology, however, are providing the most promising strategies among the third‐generation BNCT agents [30]. Nanocarriers and boron‐dense nanomaterials are being engineered to enhance tumor targeting, improve cellular uptake, and increase boron payloads, thereby addressing many of the limitations of traditional boron agents and opening new avenues for the clinical success of BNCT [31, 32, 33].

Boron‐rich structures, including polyhedral boranes, carboranes, and metallacarboranes, have exhibited promising properties in drug design [34, 35, 36, 37], and their combination with nanotechnology has significantly broadened their potential applications [38]. These boron clusters are 3D‐aromatic systems [39, 40] with exceptional chemical, thermal, and photochemical stability, which makes them outstanding candidates in materials science [41, 42, 43, 44]. In addition, these clusters offer versatile functionalization at their vertices, allowing selective modification as its reactivity depends on their position in the polyhedral framework [34, 35, 36, 37, 45].

The anionic boron cluster cobaltabisdicarbollide or **COSAN** ([Co(C_2_B_9_H_11_)_2_]^−^) [46, 47, 48, 49] exhibits unique physicochemical features including low nucleophilicity, delocalized charge density [50], and intrinsic hydrophobicity [51, 52]. These properties confer remarkable amphiphilic behavior to its protonated form and sodium salts, enabling high solubility in both aqueous and organic media (e.g. water, THF, chloroform) [53, 54, 55, 56] and self‐assembly into micelles, vesicles, and monolayer structures in aqueous solutions [57, 58, 59]. COSAN’s amphiphilic nature facilitates membrane permeability crossing efficiently the lipid bilayers without requiring transporters, cellular uptake across diverse cell types, including cancer cells, and the formation of stable nanostructures for drug delivery [60, 61, 62, 63, 64]. Besides, the *closo*‐dodecaborate anion ([B_12_H_12_]^2−^) also exhibits exceptional stability and chemical versatility that make it applicable in different fields including biomedicine, supramolecular chemistry, sustainable energy, catalysis, and optoelectronic materials [65]. Recent advances have also revealed the remarkable superchaotropic behavior of derivatives from the highly symmetrical icosahedral *closo*‐dodecaborate anion [66] and the COSAN [67]. These superchaotropic anions facilitate the translocation of hydrophilic cargos across lipid bilayers without compromising membrane integrity, offering a compelling alternative to traditional amphiphilic transporters.

High‐boron‐content systems have been widely explored by conjugating neutral and anionic boron clusters to diverse molecular scaffolds [68, 69]. COSAN and *closo*‐dodecaborate derivatives, for instance, have been covalently attached to organic platforms as dendrimers [70, 71, 72, 73], porphyrins, and phthalocyanines [74, 76]; inorganic matrices as octasilsesquioxanes [77, 78], TiO_2_ [79], and nanomaterials as Au NPs [80, 81], polymer‐based nanostructures [60, 82], single‐wall carbon nanotubes (SWCNTs) [83], and graphene oxide [84, 85], among others. These hybrids exhibit tunable physicochemical properties, including enhanced solubility, dispersibility, thermal stability, and electrochemical performance. Notably, they can also improve cellular uptake and facilitate controlled intracellular boron release. Combined with their inherently high boron density, these features position boron clusters as promising candidates for biomedical applications, particularly in BNCT [34, 35, 36, 37, 61, 86, 87].

In this work, we explore the design and potential of fullerene–boron cluster conjugates as boron carriers for next‐generation agents for BNCT. Unlike many nanomaterials, whose boron loading vary between batches, the fullerene‐based platform is a discrete molecular nanostructure with a precisely defined boron content. Furthermore, compared to most traditional dendrimers, which typically require laborious synthetic steps and complex purification protocols, fullerenes offer a more accessible route. This modular synthetic strategy can also facilitate further chemical diversification by incorporating other functionalities.

By integrating the multivalent and modular scaffold of [60]fullerene, our aim is to establish a versatile strategy to describe new boron‐dense molecular materials that could address key challenges in BNCT. To the best of our knowledge, this study represents the first report on hybrids that combine fullerenes with anionic boron clusters, thereby opening an unexplored avenue for the development of multifunctional pharmacophores in cancer therapy.

## 2 Results and Discussion

### 2.1 Synthetic Methods and Characterization

We have considered the decoration of [60]fullerene with COSAN and [B_12_H_12_]^2−^ anionic boron clusters following a Cu(I)‐catalyzed Azide–Alkyne Cycloaddition (CuAAC) “click” methodology [15]. This was achieved synthetically through the appropriated functionalization of C_60_ and the corresponding boron clusters suitable to be clicked. On one hand, C_60_ hexa‐adduct (**1**) bearing twelve alkynyl‐terminated moieties was synthesized via Bingel–Hirsch cyclopropanation reaction [88]. On the other hand, oxonium derivatives of COSAN and [B_12_H_12_]^2−^ were chosen for their versatility in undergoing ring‐opening reactions with various nucleophiles [89, 90, 91]. Their corresponding azides [8‐N_3_‐C_4_H_8_O_2_‐3,3′‐Co(1,2‐C_2_B_9_H_10_)(1′,2′‐C_2_B_9_H_11_)]^−^ (**2**) and [1‐(N_3_‐C_4_H_8_O_2_)‐B_12_H_11_]^2−^ (**3**) were obtained by ring‐opening reaction using NaN_3_ as nucleophile [92].

Therefore, to attach twelve boron clusters to hexa‐adduct **1**, reactions were carried out in DMSO solutions using CuBrꞏSMe_2_ and Cu(0) as catalytic system. Moreover, 33% excess of boron cluster derivatives **2** or **3** were added to ensure the total functionalization of **1**. After completion of the reactions, copper traces were removed by a Quadrasil MP to avoid any metal‐derived toxicity. Finally, purification through column chromatography afforded pure polyanionic **C**_**60**_ **–COSAN** in 53% yield, whereas **C**_**60**_ **–B**_**12**_ was isolated in 60% yield by precipitation with [NBu_4_]Br (Figure 1).

**FIGURE 1:**
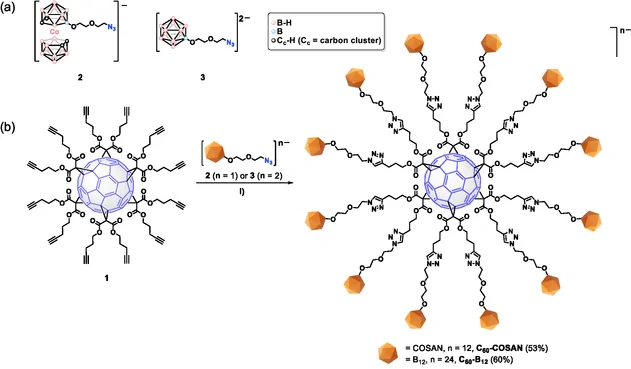
Synthesis of boron‐rich fullerene cycloadducts. (a) Azide derivatives of COSAN (**2**) and ([B_12_H_12_]^2−^ (**3**) used to obtain (b) **C**_**60**_ **–COSAN** and **C**_**60**_ **–B**_**12**_, respectively. Reagents and conditions: (i) CuBr·S(CH_3_)_2_, Cu^0^, sodium ascorbate, DMSO, r.t., 72 h.

The molecular structure of target compounds was confirmed by NMR experiments (^1^H, ^13^C, ^11^B, ^11^B{^1^H}) as well as IR‐ATR spectroscopy. The ^1^H NMR spectra provide a straightforward way to establish that *click* reaction took place, which is the appearance of the expected triazole linkage resonances near 8 ppm for both **C**_**60**_ **–COSAN** and **C**_**60**_ **–B**_**12**_ (Figures S1 and S6). In the former, the presence of COSAN is confirmed by two resonances at *δ* 4.20 and 4.18 ppm unequivocally attributed to the protons linked to the carbon cluster (C_c_–H), while both compounds display the characteristic broad band of B–H protons in the range 0–3 ppm. The ^13^C{^1^H} NMR (Figures S2 and S7) easily provides evidence for the octahedral substitution pattern of [60]fullerene hexa‐adducts, showing only two resonances at *δ *≈ 142 and 147 ppm attributed to C_sp_^2^ of C_60_ (Figure 2). These spectra also confirmed the formation of the target compounds: firstly, the terminal alkynyl carbon resonances of **1** at ~90–70 ppm (─C≡CH) are no longer present; secondly, **C**_**60**_ **–COSAN** and **C**_**60**_ **–B**_**12**_ show two new signals around *δ* 124 and 146 ppm corresponding to the triazole ring. Again, resonances from C_cluster_ (C_c_) are found in **C**_**60**_ **–COSAN** spectrum near 47 and 55 ppm.

**FIGURE 2:**
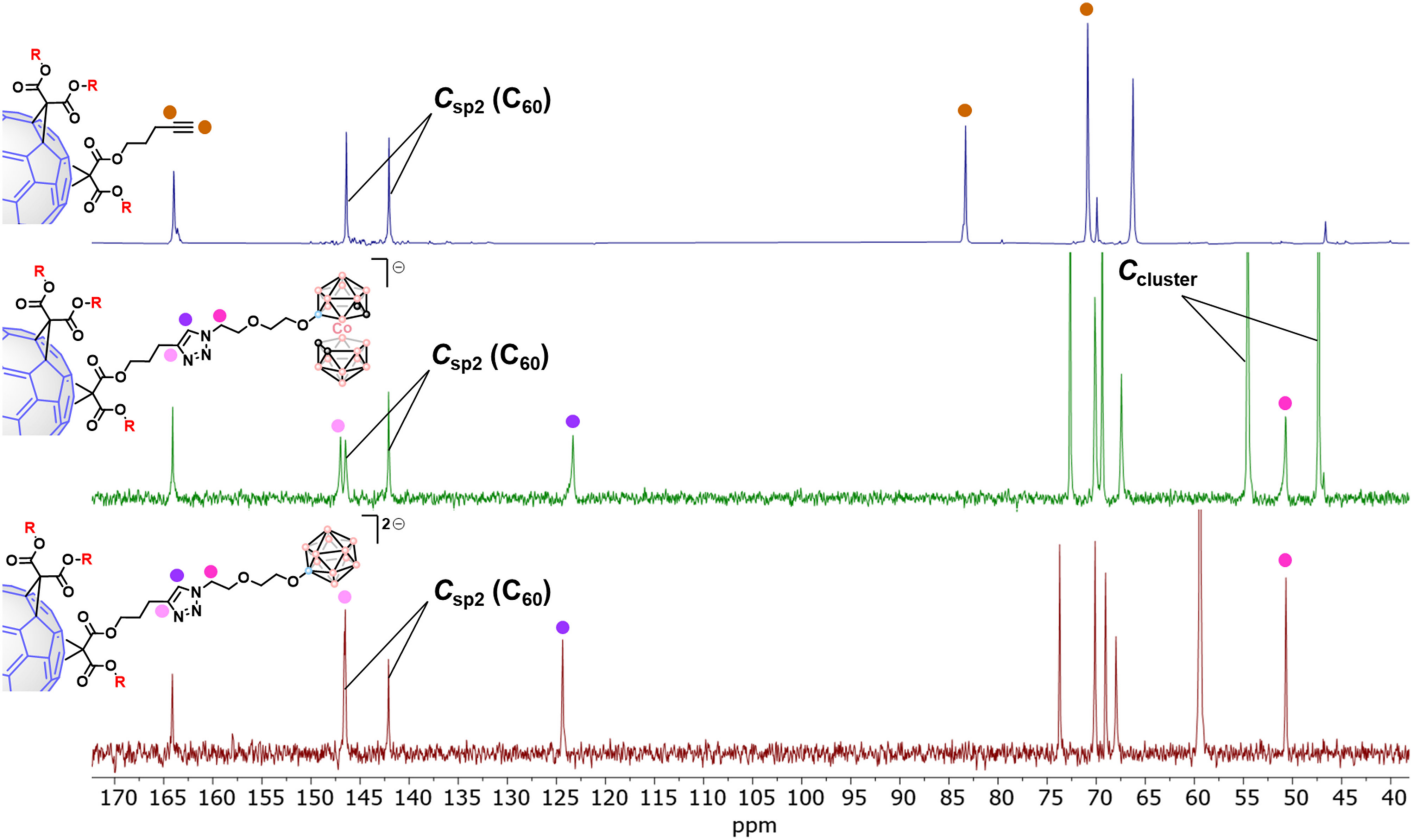
^13^C NMR spectra of **1** (up), **C**_**60**_ **–COSAN** (middle) and **C**_**60**_ **–B**_**12**_ (down) in acetone‐*d*_6_.

The ^11^B{^1^H} NMR spectra proved highly valuable to confirm that both boron clusters keep their structural integrity under CuAAC conditions as well as the successful functionalization of C_60_: **C**_**60**_ **–COSAN** shows the typical 1:1:1:1:2:3:3:2:2:1:1 pattern of oxonium derivatives of COSAN [62], while a 1:5:5:1 distribution is observed for **C**_**60**_ **–B**_**12**_ (Figures S3 and S8) [93]. In both cases, the lowest field resonance is assigned to the boron atom from the substituted B–O vertex. The IR‐ATR spectra clearly point out the presence of boron clusters (Figures S5 and S10). Starting derivatives **2**–**‍3** and target nanostructures show the typical ʋ(B−H) strong bands below 2500 cm^−1^ for those bearing *closo*‐dodecaborate and over 2500 cm^−1^ for those bearing COSAN. Moreover, the intense ʋ(N═N═N) stretching bands of the azide groups in **2** and **3** spectra are absent in **C**_**60**_ **–COSAN** and **C**_**60**_ **–B**_**12**_, confirming that any excess of starting clusters **2**‐**3** was removed through the purification of the final nanomaterials.

The solution‐phase purity and monodispersity of **C**_**60**_ **−COSAN** and **C**_**60**_ **−B**_**12**_ were confirmed by DOSY NMR experiments (Figures S13 and S14). All resonances assigned to the adducts exhibited a uniform diffusion coefficient (*D*), proving the presence of a single molecular entity with no observable impurities. Furthermore, the calculated hydrodynamic radii *R*_H_ ≈ 2 nm for both hybrids align with the expected dimensions for these discrete fullerene hexakis‐adduct nanostructures (Table S1) [94].

### 2.2 UV–Vis Absorption and Estimation of COSAN Units

Steady‐state UV–Vis absorption measurements were performed in acetonitrile for all compounds: **1**–**3**, **C**_**60**_ **–COSAN,** and **C**_**60**_ **–B**_**12**_ (Figures 3 and S11).

**FIGURE 3:**
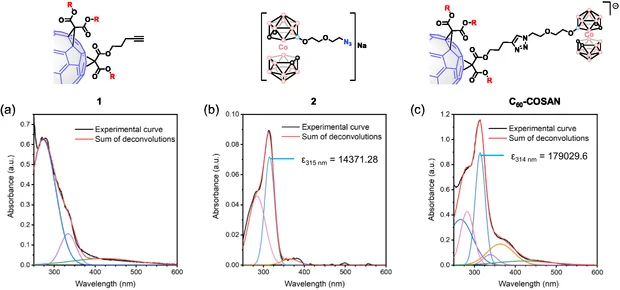
UV–Vis spectra in CH_3_CN of (a) compound **1** (1 · 10^−6^ M), (b) compound **2** (5 · 10^−6^ M) and (c) **C**_**60**_ **–COSAN** (5 · 10^−6^ M) and its corresponding gaussian deconvolution curves.

The absorption spectra of starting C_60_ derivative **1** shows a broad absorption band below 350 nm, with a maximum centered at 275 nm, as in other hexa‐adducts of fullerene with similar substitution patterns [95]. Moreover, boron cluster derivatives **2**–**3** also show expected UV–Vis spectra: compound **2** displays two maxima near 310 and 375 nm endorsed to the metallacarborane unit [77, 96], while compound **3** does not show any absorption band in the UV–Vis spectrum as previously observed for *closo*‐dodecaborate compounds [78, 97]. Then, it is expected that UV–Vis spectra of final nanohybrids show the contribution of each moiety, *i.e*., the C_60_ hexa‐adduct core and the boron clusters. In this regard, **C**_**60**_ **–COSAN** displays those COSAN bands described for **2** and a shoulder around 300 nm ascribed to the fullerene core. However, absorption spectra of starting hexa‐adduct **1** and final **C**_**60**_ **–B**_**12**_ are very much alike, which is due to the negligible contribution of *closo*‐dodecaborate to the UV–Vis.

In addition, UV–Vis measurements acquired in the same solvent can be used to estimate the units of a chromophore attached to different scaffolds (Figure 3). This method proposed by Kim and Jung [98] to quantify phenyl‐ethynyl moieties in dendrimers has proven effective for estimating the number of COSAN clusters in various multivalent constructions [77, 99]. Gaussian deconvolution of the absorption spectra enables the identification of bands corresponding to COSAN units, typically observed with *λ*_max_ = 314–315 nm (Figure 3). By comparing the molar absorptivity of compound **2** (*ε*_315_), which contains a single COSAN unit, with that of **C**_**60**_ **–COSAN** (*ε*_314_), which bears 12 peripheral COSAN clusters, a ratio of 1:12.4 is obtained. This value closely matches the expected number of metallacarborane units in the hybrid structure, thus validating the approach.

### 2.3 Electrochemical Characterization

Finally, considering the reversible redox electroactivity of metallacarboranes, cyclic voltammetry (CV) measurements were carried out to confirm the presence of COSAN in **C**_**60**_ **–COSAN** (Figure ‍S12). Both compounds **2** and **C**_**60**_ **–COSAN** exhibit the same reversible redox process around −1.75 V versus Ag/Ag^+^, attributed to the Co^3+^/Co^2+^ redox transition, in agreement with previous reports [42]. In **C**_**60**_ **–COSAN,** additional redox processes are observed due to the presence of the fullerene core. Two quasi‐reversible reduction waves appear at −1.15 and −1.29 V, which are negatively shifted compared to pristine C_60_. This shift is attributed to the saturation of double bonds in the fullerene framework of the hexa‐adduct [100]. Together, these findings confirm that the redox signatures of both metallacarborane and fullerene components are preserved in the multivalent hybrids. Furthermore, in both compounds **3** and **C**_**60**_ **–B**_**12**_, the *closo*‐dodecaborate moiety undergoes an oxidation process around +0.7 V [42]. This oxidation, though typically irreversible, further supports the successful incorporation of the boron‐rich cluster onto the fullerene scaffold and its retention of electrochemical activity.

### 2.4 Physicochemical Characterization of Liposomal Formulations for Biological Assays

To enable efficient cellular delivery of the hydrophobic C_60_‐based compounds (**C**_**60**_ **–COSAN** and **C**_**60**_ **–B**_**12**_), the molecules were incorporated into liposomes, which were employed as biocompatible nanocarriers to enhance solubility, stability, and bioavailability. Liposomes were formulated from POPC/Chol/DSPE‐PEG2000/RhPE at a molar ratio of 85/5/10/0.5 (mol/mol/mol/mol), where cholesterol was included to improve membrane stability and DSPE‐PEG2000 to enhance colloidal stability and cellular compatibility. Rhodamine‐PE (RhPE) was incorporated as a fluorescent probe to enable visualization and tracking of the liposomes by confocal microscopy.

Prior to the biological evaluation, the physicochemical properties of the liposomal formulations were characterized to confirm their suitability as nanocarriers. The hydrodynamic diameter and polydispersity index (PDI) were determined by dynamic light scattering (DLS), whereas the surface charge was evaluated by zeta potential measurements. Encapsulation efficiency (EE) and drug loading (DL) were quantified by UV–Vis absorption spectroscopy at 276 and 314 nm for liposome‐encapsulated **C**_**60**_ **–COSAN** and **C**_**60**_ **–B**_**12**_, respectively (Figure S11), after separation of the nonencapsulated compound by ultracentrifugation. The absorbance of both compounds at the selected wavelengths was sufficiently high to minimize any contribution from light scattering by the liposomal suspensions.

As summarized in Table 1, unloaded liposomes exhibited an average hydrodynamic diameter of 88.5 ± 4.3 nm, a PDI of 0.22 ± 0.02, and a zeta potential of −8.2 ± 0.5 mV. Incorporation of the boron‐rich hybrids increased the average particle size to 228.0 ± 20.0 nm for **C**_**60**_ **–B**_**12**_‐loaded liposomes and 186.9 ± 9.4 nm for **C**_**60**_ **–COSAN**‐loaded liposomes, with PDI values of 0.55 ± 0.05 and 0.54 ± 0.05, respectively. The corresponding zeta potentials were −29.3 ± 1.8 mV for liposome‐encapsulated **C**_**60**_ **–B**_**12**_‐loaded liposomes and −11.0 ± 0.7 mV for **C**_**60**_ **–COSAN**‐loaded liposomes. The more negative surface charge of the **C**_**60**_ **–B**_**12**_ formulation is consistent with its higher anionic character.

**TABLE 1:** Physicochemical characterization of liposome‐encapsulated **C**_**60**_ **–COSAN** and **C**_**60**_ **–B**_**12**_.

Formulation	Size, nm	PDI	Zeta potential, mV	EE, %	DL, %
Liposomes	88.5 + −4.3	0.22 + −0.02	−8.2 + −0.5	—	—
C_60_–B_12_ liposomes	228.0 ± 20.0	0.55 ± 0.05	−29.3 ± 1.8	68.4 ± 4.0	25.5 ± 1.5
C_60_–COSAN liposomes	186.9 ± 9.4	0.54 ± 0.05	−11.0 ± 0.7	68.7 ± 4.1	8.0 ± 0.5

*Note:* Values are expressed as mean ± SD (*n* = 3 technical replicates). Hydrodynamic diameter and PDI were determined by dynamic light scattering (DLS), zeta potential by electrophoretic light scattering, and encapsulation efficiency (EE) and drug loading (DL) by UV–Vis spectroscopy after separation of nonencapsulated compound.

Both formulations exhibited comparable encapsulation efficiencies of 68.4% ± 4.0% for liposome‐encapsulated **C**_**60**_ **–B**_**12**_‐loaded liposomes and 68.7% ± 4.1% for **C**_**60**_ **–COSAN**‐loaded liposomes, indicating a similar encapsulation performance. DL values were 25.5 ± 1.5% for liposome‐encapsulated **C**_**60**_ **–B**_**12**_ and 8.0% ± 0.5% for liposome‐encapsulated **C**_**60**_ **–COSAN**. The higher DL obtained for the **C**_**60**_ **–B**_**12**_ formulation reflects the larger amount of hybrid used during liposome preparation, which was selected to achieve comparable boron concentrations in the biological assays while accounting for the different boron contents of the two fullerene–boron hybrids. Overall, these results confirm the successful incorporation of both boron‐rich hybrids into the liposomal carriers and support their subsequent evaluation in the in vitro biological assays.

### 2.5 Colloidal Stability and Cargo Retention of Liposomal Formulations

To evaluate the stability of the liposomal formulations under physiologically relevant conditions, unloaded and loaded liposomes were incubated at 37 °C in 100 mM HEPES buffer (pH 7.4), mimicking the extracellular physiological environment, 10 mM MES buffer (pH 5.0), representative of the acidic endolysosomal compartment following cellular uptake, or complete cell culture medium to assess their behavior in the presence of serum proteins.

Again, changes in hydrodynamic diameter were monitored by DLS for up to 72 h, whereas the amount of encapsulated hybrid remaining after incubation was determined by UV–Vis spectroscopy following ultracentrifugation for samples incubated in HEPES and MES buffers. Note that quantification of the encapsulated compounds after incubation in complete culture medium was not feasible because serum proteins and other medium components generated significant background absorption, preventing reliable spectroscopic determination of the boron‐rich hybrids.

As summarized in Table 2, unloaded liposomes remained colloidally stable over the 72 h incubation period, exhibiting only minor variations in hydrodynamic diameter (88.5–84.8 nm). Similarly, both boron‐loaded formulations preserved their nanoscale dimensions under neutral and mildly acidic conditions. **C**_**60**_ **–COSAN**‐loaded liposomes showed only modest changes in particle size in both HEPES and MES buffers, whereas **C**_**60**_ **–B**_**12**_‐loaded liposomes exhibited a gradual decrease in hydrodynamic diameter during incubation. Nevertheless, no evidence of extensive aggregation or precipitation was observed under either condition. Measurements performed in complete culture medium displayed larger fluctuations in hydrodynamic diameter for both formulations, which are attributed to interactions between serum proteins and the liposomal surface, leading to protein corona formation and the increased complexity of DLS analysis in biological media.

**TABLE 2:** Colloidal stability and cargo retention of unloaded and boron‐loaded liposomes under physiologically relevant conditions.

Formulation	Initial size, nm	Size, nm after 24 h	Size, nm after 48 h	Size, nm after 72 h	Cargo retention after 72 h, %
Liposomes	88.5 ± 4.3	87.6 ± 4.3	86.0 ± 4.2	84.8 ± 4.2	—
**C**_**60**_ **–B**_**12**_ liposomes (pH 7.4)	228.0 ± 20.0	175.3 ± 15.9	96.66 ± 9.5	130.9 ± 12.9	82
**C**_**60**_ **–B**_**12**_ liposomes (pH 5)	207.1 ± 10.5	209.8 ± 10.2	169.1 ± 9.1	162.3 ± 9.0	92
**C**_**60**_ **–B**_**12**_ liposomes (in culture medium)	108.5 ± 10.5	106.4 ± 10.4	165.9 ± 15.9	144.8 ± 15.0	—
**C**_**60**_ **–COSAN** liposomes (pH 7.4)	186.9 ± 9.4	186.6 ± 9.4	169.4 ± 9.0	172.7 ± 9.0	87
**C**_**60**_ **–COSAN** liposomes (pH 5)	169.2 ± 8.5	168.3 ± 8.5	158.2 ± 8.4	160.1 ± 8.5	88
**C**_**60**_ **–COSAN** liposomes (in culture medium)	141.2 ± 14.2	158.8 ± 15.0	106.7 ± 10.4	108.1 ± 10.5	—

*Note:* Hydrodynamic diameter was determined by dynamic light scattering (DLS) immediately after preparation (0 h) and after incubation for 24, 48, and 72 h at 37 °C in 100 mM HEPES buffer (pH 7.4), 10 mM MES buffer (pH 5.0), or complete cell culture medium. Initial polydispersity index (PDI), zeta potential, and encapsulation efficiency (EE) are also shown. Cargo retention after 72 h was determined by UV–Vis spectroscopy following ultracentrifugation and is expressed as the percentage of the initially encapsulated compound remaining associated with the liposomes. Quantification after incubation in complete culture medium was not performed because serum proteins and medium components interfered with the UV–Vis determination. Values are expressed as mean ± SD (*n* = 3).

Retention of the encapsulated boron‐rich hybrids after 72 h remained high in both buffer systems. **C**_**60**_ **–COSAN**‐loaded liposomes retained **87%** and **88%** of the initially encapsulated compound after incubation in HEPES (pH 7.4) and MES (pH 5.0), respectively. **C**_**60**_ **–B**_**12**_‐loaded liposomes retained **82%** and **92%** under the same conditions. These results indicate that both formulations maintain a substantial fraction of their encapsulated cargo under conditions that mimic the extracellular and lysosomal environments, supporting their suitability as boron delivery systems for the subsequent biological studies.

### 2.6 Biological Assays

Based on their favorable physicochemical characteristics, satisfactory cargo retention under physiologically relevant conditions, and good colloidal stability, the liposome‐encapsulated **C**_**60**_ **–COSAN** or **C**_**60**_ **–B**_**12**_ were selected for subsequent in vitro biological evaluation. To enable visualization of the liposomal nanocarriers without chemically modifying the boron‐rich hybrids, RhPE was incorporated as a fluorescent probe (0.5% mol) to enable visualization and tracking of the liposomes by confocal microscopy. Mouse embryonic fibroblasts (MEFs) were utilized to study compound internalization because they provide a well‐characterized cellular model and robust endocytic activity.

Prior to assessing liposome internalization in MEFs, cytocompatibility was evaluated at a final liposome concentration of 0.3 or 0.2 mg/mL for **C**_**60**_ **–COSAN** and **C**_**60**_ **–B**_**12**_, respectively. Both C_60_‐based compounds were tested over a range of concentrations and showed no detectable cytotoxicity up to the highest boron concentration evaluated (26 µg B/mL) (Figure 4a). Upon confirmation of biocompatibility, cellular uptake and intracellular trafficking were investigated. MEFs were incubated with liposome‐encapsulated **C**_**60**_ **–COSAN** or **C**_**60**_ **–B**_**12**_ at a concentration of 10 µg B/mL for up to 48 h. The 48 h incubation period was selected because both formulations remained colloidally stable and retained most of their encapsulated cargo under physiologically relevant conditions. Confocal microscopy revealed a punctate intracellular fluorescence pattern, with discrete fluorescent spots distributed throughout the cytoplasm, consistent with efficient internalization of the liposomes into vesicular structures (Figure 4b).

**FIGURE 4:**
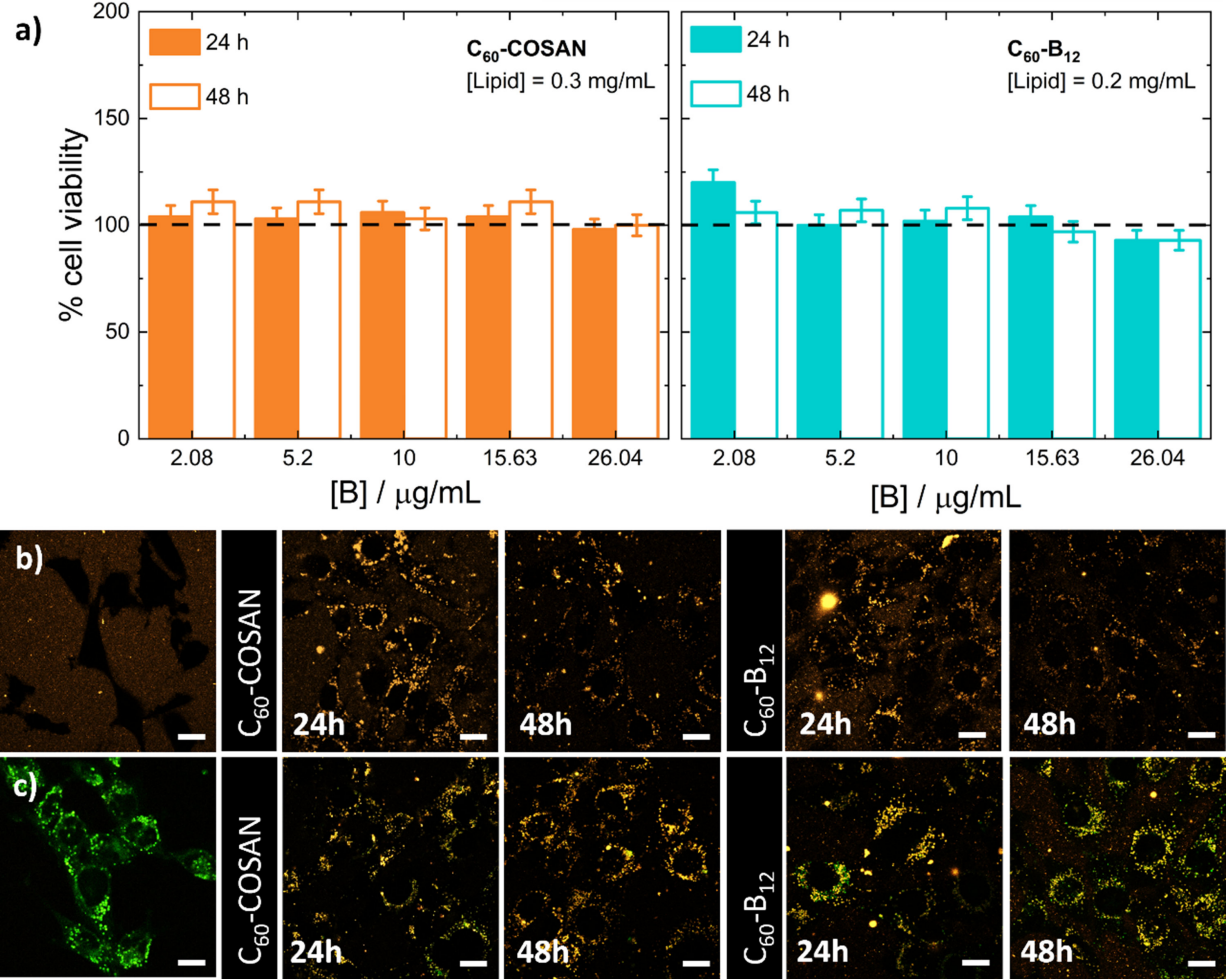
(a) MEF viability after addition of liposome‐encapsulated **C**_**60**_ **–COSAN** (left) and **C**_**60**_ **–B**_**12**_ (right), containing different boron concentration. Mean and standard deviation were calculated from 3 technical replicates per point. The dotted line in both graphs represents 100% cell viability, corresponding to the untreated control cells. (b) Confocal fluorescence micrographs of MEFs incubated with 0.3 and 0.2 mg/mL of liposome‐encapsulated **C**_**60**_ **–COSAN** and **C**_**60**_ **–B**_**12**_ (final boron concentration: 10 µg/mL). MEFs were imaged after 24 h (left) and 48 h (right) of incubation. Rhodamine‐PE (RhPE) was incorporated as a fluorescent probe to enable visualization and tracking of the liposomes by confocal microscopy. (c) Merged green‐ and red‐channel confocal fluorescence micrographs of living MEFs costained with LysoTracker Green (1 μM) and liposome‐encapsulated **C**_**60**_ **–COSAN** or **C**_**60**_ **–B**_**12**_, respectively (final boron concentration: 10 µg/mL). Cells were imaged after 24 h (left) and 48 h (right) of incubation. Green channel: *λ*_exc_ = 488 nm and *λ*_em_ = 525 ± 25 nm. Red channel: *λ*_exc_ = 561 nm and *λ*_em_ > 561 nm. Scale bars are 10 μm.

To assess subcellular localization, cells were then coincubated with LysoTracker Green, a selective fluorescent marker for acidic compartments such as late endosomes and lysosomes. Confocal fluorescence imaging was performed at 0, 24, and 48 h, and colocalization analysis was conducted by overlaying the RhPE (red) and LysoTracker Green (green) channels. The appearance of yellow fluorescence in merged images indicated significant colocalization (Figure 4c), suggesting that the internalized liposomes predominantly trafficked through endolysosomal pathways.

Once biocompatibility and internalization were confirmed in MEFs, the effect of liposome‐encapsulated **C**_**60**_ **–COSAN** or **C**_**60**_ **–B**_**12**_ on tumor cells was investigated to gain a better understanding of their potential use in BNCT. The Cal33 cell line—derived from a human head and neck tumor—was selected as this type of tumor is one of the current targets of this therapy. Cytotoxicity was evaluated over a 48‐h period across a range of concentrations. As shown in Figure 5a, none of the compounds (**C**_**60**_ **–COSAN** or **C**_**60**_ **–B**_**12**_) exhibited significant cytotoxicity, with only a minor reduction in cell viability observed at a concentration of 26 µg B/mL. Cellular uptake and intracellular localization were further examined by confocal microscopy and coincubation with LysoTracker Green. Short‐term incubation (2 h) confirmed rapid internalization of both compounds and suggested that **C**_**60**_ **–COSAN** and **C**_**60**_ **–B**_**12**_ are internalized predominantly via endocytic pathways (Figure ‍5b). Colocalization with lysosomal markers provided evidence of successful uptake and indicated a potential degradative trafficking route within the lysosomal compartment.

**FIGURE 5:**
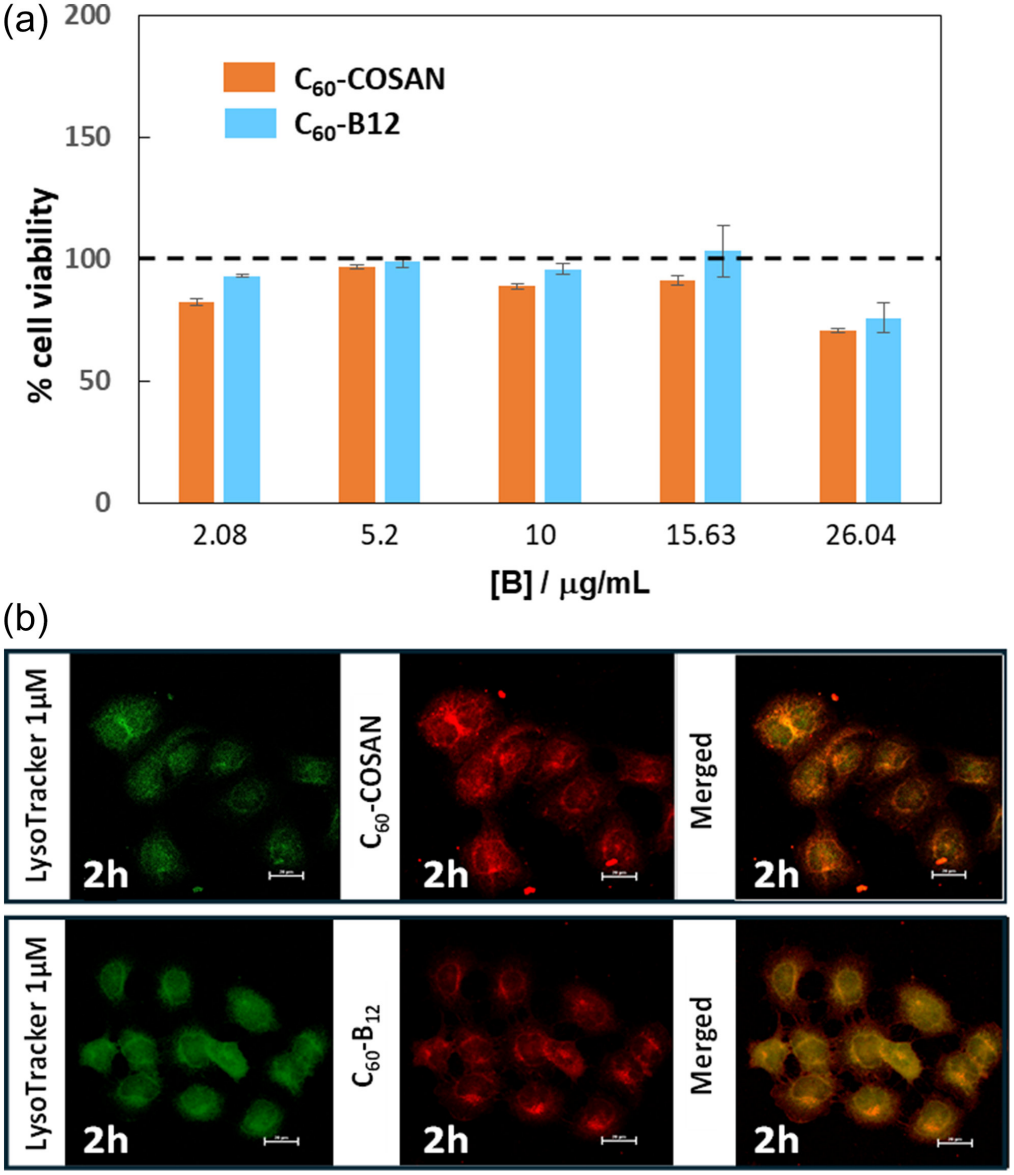
(a) Viability of Cal33 cells after 48 h of incubation with liposome‐encapsulated **C**_**60**_ **–COSAN** and **C**_**60**_ **–B**_**12**_ containing different boron concentrations. Mean and standard deviation were calculated from 3 replicates per point. The dotted line represents 100% cell viability, corresponding to the untreated control cells. (b) Confocal fluorescence microscopy images of live Cal33 cells incubated with **C**_**60**_ **–COSAN** and **C**_**60**_ **–B**_**12**_ at a concentration of 10 μg B/mL. Rhodamine‐PE (RhPE) was incorporated as a fluorescent probe to the liposomes and cells were costained with LysoTracker Green (1 μM) as lysosomal marker. The images (green, red, and merged green and red channels) were taken after 2 h of incubation. Green channel: *λ*_exc_ = 488 nm and *λ*_em_ = 525. Red channel: *λ*_exc_ = 561 nm and *λ*_em_ > 561 nm. Scale bars are 20 μm.

## 3 Conclusions

In this work, we have developed a new class of boron‐rich nanomaterials based on the covalent integration of anionic boron clusters onto a multivalent [60]fullerene scaffold. By exploiting a Cu(I)‐catalyzed azide–alkyne CuAAC strategy, [60]fullerene hexakis‐adducts bearing twelve peripheral COSAN or *closo*‐dodecaborate units were successfully synthesized, yielding well‐defined polyanionic hybrids with exceptionally high boron payloads. Comprehensive spectroscopic and electrochemical characterization confirmed the structural integrity of both the fullerene core and the boron clusters after functionalization.

To enable preliminary biological evaluation, the fullerene–boron cluster conjugates were efficiently incorporated into liposomal formulations, which provided good aqueous dispersibility and biocompatibility. The resulting nanocarriers showed negligible cytotoxicity and efficient cellular uptake in both MEFs and Cal33 human head and neck cancer cells, with predominant localization in endolysosomal compartments. These results suggested that high intracellular boron concentrations were achieved without compromising cell viability under the conditions tested.

These results establish fullerene–boron cluster hybrids as a versatile and modular platform for the construction of high‐boron‐content nanomaterials. As the first example of conjugates combining fullerenes with anionic boron clusters, this work opens new opportunities for the development of advanced boron delivery agents. We would also like to emphasize that, while neutron irradiation studies are beyond the scope of this present work, the revised manuscript successfully demonstrates several key prerequisites for prospective BNCT delivery systems: high boron payloads, robust liposomal formulation, colloidal stability under physiologically relevant conditions, negligible cytotoxicity, and cellular uptake in a clinically relevant head and neck cancer cell line. These findings provide an experimental basis foundation for future neutron irradiation studies.

## Funding

This study was supported by the Spanish Ministry of Science, Innovation, and Universities MICIU/AEI/10.13039/501100011033 through projects (PID2022‐136892NB‐I00, PID2023‐148420NB‐I00, and PID2023‐146373OB‐I00) and Spanish Agencia Estatal de Investigación through the Severo Ochoa Program for Centers of Excellence in R&D (CEX2023‐001263‐S).

## Supporting information

Supplementary Material

## Data Availability

The data that support the findings of this study are available from the corresponding author upon reasonable demand.
