# Supplementary material for: [60]Fullerene–Cluster Hybrids as Boron‐Dense Delivery Nanoplatforms
Source: Small Sci. 2026 Oct 7;6(10):e70413. doi: 10.1002/smsc.70413 (PMC13646491; doi:10.1002/smsc.70413)
Supplement: Supplementary file 1 [file SMSC-6-e70413-s001.pdf]

Supporting Information

[60]Fullerene–Cluster Hybrids as Boron-Dense Delivery Nanoplatforms

Jennifer Patino-Alonso,[a] *Mónica Muñoz-Úbeda,*[b,c] *Iván López-Montero,*[b,c,d] *M. José Ruiz-Magaña,*[e] *Carmen Ruiz-Ruiz,*[e] *Ignacio Porras,*[f] *Miquel Nuez-Martínez,* [g] *Rosario Núñez,**[g] *Beatriz M. Illescas,*[a] *Justo Cabrera-González,** [a,h] *and Nazario Martín**[a,i]

[a] Departamento de Química Orgánica, Facultad de Química, Universidad Complutense, E-28040 Madrid, Spain.
[b] Departamento de Química Física, Facultad de Química, Universidad Complutense, 28040 Madrid, Spain.
[c] Instituto de Investigación Sanitaria Hospital Doce de Octubre (imas12), 28041 Madrid, Spain
[d] Instituto Pluridisciplinar, Paseo Juan XXIII, 1, 28040 Madrid, Spain
[e] Instituto de Biopatología y Medicina Regenerativa, Centro de Investigación Biomédica, Universidad de Granada, Avda. del Conocimiento s/n. 18016 Granada, Spain.
[f] Departamento de Física Atómica, Molecular y Nuclear, Facultad de Ciencias, Universidad de Granada, Avenida de Fuentenueva S/N 18071 Granada.
[g] Institut de Ciència de Materials de Barcelona (ICMAB-CSIC), Carrer dels Til.lers, 08193 Cerdanyola del Vallès, Spain.
[h] Departamento de Química en Ciencias Farmacéuticas, Facultad de Farmacia, Universidad Complutense, E-28040 Madrid, Spain.
[i] IMDEA-Nanoscience, C/ Faraday 9, Campus de Cantoblanco, 28049 Madrid, Spain.

1. Experimental Section

1.1 General Methods

Reagents and solvents were obtained from commercial suppliers and used without further purification. Compounds **1**[1], **2**[2] and **3**[3] were prepared according to previously reported procedures. All boron cluster precursors used in this work contained boron at their natural isotopic abundance. All work-up and purification procedures were carried out with HPLC-grade solvents in air. Flash chromatography was performed using Scharlab silica gel (230-400 mesh, 0.040-0.063 mm). Analytical thin layer chromatography (TLC) was performed using E. Merck silica gel 60 F_{254} precoated plates (0.25 mm). The developed chromatogram was analyzed by UV lamp (λ = 254 nm). ^{1}H, ^{11}B and ^{13}C NMR spectra were performed on a Bruker AVIII HD 300MHz BACS-60 (^{1}H: 300

MHz, ^{13}C: 75 MHz), Bruker AV 500MHz (^{1}H: 500 MHz, ^{13}C: 126 MHz, ^{11}B: 160 MHz) and Bruker AVIII 700MHz (^{1}H: 700 MHz, ^{13}C: 176 MHz) at 298 K using partially deuterated solvents as internal standards. Coupling constants (*J*) are expressed in Hertz (Hz) and chemical shifts (δ) in parts per million (ppm) relative to the solvent. Data are reported as follows: s = singlet, t = triplet, sx = sextet, m = multiplet. IR spectra (cm^{-1}) were recorded on a Spectrum 3™ FT-IR Spectrometer. Data are reported as follows: s = strong, m = medium, w = weak. UV-Vis absorption spectra were performed in a Shimadzu UV-3600 UV-VIS-NIR Spectrophotometer, using 1 cm pathlength quartz cells at 298 K. Cyclic voltammetry (CV) experiments were recorded in a Potentiostat Autolab PGSTAT 30 electrochemical analyzer. All measurements were performed at 298 K under argon atmosphere in 0.1 M solutions of tetrabutylammonium chloride (TBACl) in CH_3CN. A three-electrode configuration was used throughout, using glassy carbon as working electrode, platinum wires were used as counter electrode and Ag/0.1 M $AgNO_3$ as reference electrode. All redox potentials are referenced against ferrocene/ferrocenium couple (Fc/Fc^+) as the internal standard.

1.2. Synthetic procedures

C_{60}-COSAN

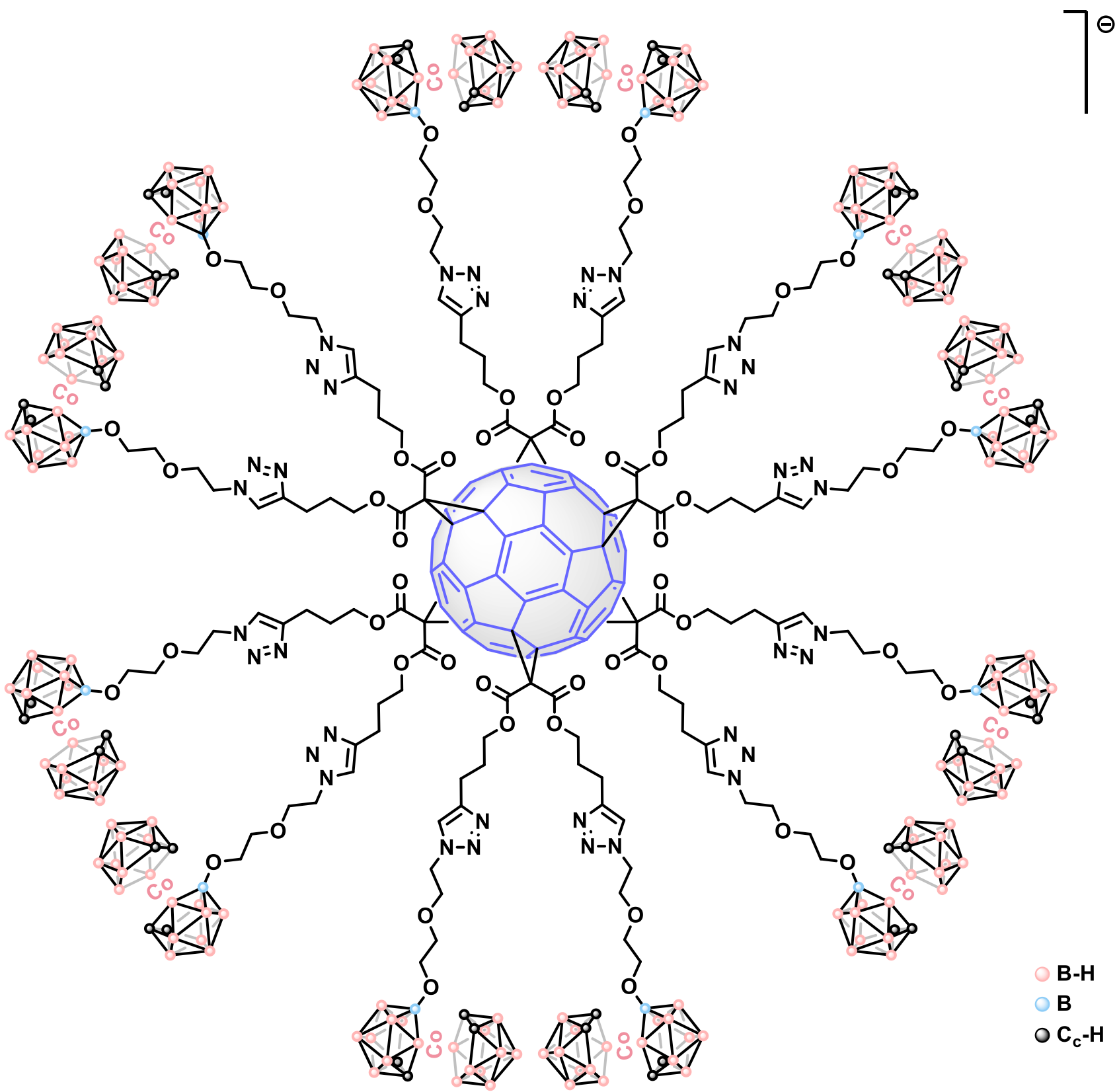

A mixture of compound **1** (20 mg, 9.4 μmol), compound **2** (72 mg, 0.15 mmol), $CuBr{\cdot}S(CH_3)_2$ (16 mg, 0.075 mmol), sodium ascorbate (30 mg, 0.15 mmol) and a piece of copper metal wire in DMSO (1 mL) was deoxygenated and kept under argon with vigorous stirring for 72 h. The crude reaction was filtered through a Quadrasil® Mercaptopropyl column and purified by column chromatography

(SiO_2, CH_2Cl_2 → CH_2Cl_2/CH_3CN 3:1 → CH_3CN) to obtain **C_{60}-COSAN** as a red glassy solid (39 mg, 53%). **^{1}H NMR** (700 MHz, acetone-d_6) δ: 7.82 (s, 12H; C$H_{triazole}$), 4.58 – 4.52 (m, 24H; CH_2N), 4.49 – 4.36 (m, 24H; COOCH_2), 4.21 – 4.17 (m, 48H; $C_{cluster}$-H), 3.92 – 3.87 (s, 24H; CH_2O), 3.63 – 3.60 (m, 24H; CH_2O), 3.56 – 3.53 (s, 24H; CH_2O), 2.87 – 2.74 (m, 24H; $COOCH_2CH_2CH_2$), 2.09 (m, 24H; $COOCH_2CH_2CH_2$ overlaps with acetone); **^{13}C NMR** (176 MHz, acetone-d_6) δ: 164.1 (*C*O), 146.9 ($C_{sp2\ fullerene}$), 146.5 ($C_{triazole}$), 142.1 ($C_{sp2\ fullerene}$), 123.3 ($CH_{triazole}$), 72.6, 70.1 (CH_2O), 69.4 (CH_2O and $C_{sp3\ fullerene}$), 67.4 ($COOCH_2$), 54.6 ($C_{cluster}$-H), 50.7 (CH_2N), 47.3 ($C_{cluster}$-H), 29.0 ($COOCH_2CH_2CH_2$), 22.6 ($COOCH_2CH_2CH_2$); **^{11}B{^{1}H} NMR** (160 MHz, acetone-d_6) δ: 23.32 (s, 12B; *B*O), 4.46 (s, 24B; *B*H), 0.21 (s, 24B; *B*H), -2.64 (s, 24B; *B*H), -4.62 (s, 24B; *B*H), -8.12 (s, 36B; *B*H), -17.55 (s, 24B; *B*H), -20.64 (s, 24B; *B*H), -28.79 (s, 24B; *B*H); **^{11}B NMR** (160 MHz, acetone-d_6) δ: 23.32 (s, 12B; *B*O), 4.48 (d, $^1J_{B,H}$ = 131.1 Hz, 24B; *B*H), 0.25 (d, $^1J_{B,H}$ = 123.1 Hz, 24B; *B*H), -2.68 (d, $^1J_{B,H}$ = 125.4 Hz, 24B; *B*H), -4.42 (d, $^1J_{B,H}$ = 126.9 Hz, 24B; *B*H), -6.12 – -13.01 (m, 36B; *B*H), -17.49 (d, $^1J_{B,H}$ = 139.3 Hz, 24B; *B*H), -20.60 (d, $^1J_{B,H}$ = 155.6 Hz, 24B; *B*H), -29.16 (d, $^1J_{B,H}$ = 151.6 Hz, 24B; *B*H). **FTIR-ATR** (ν cm^{-1}) = 2947 (w, CH), 2560 (s, BH), 1740 (m, CO).

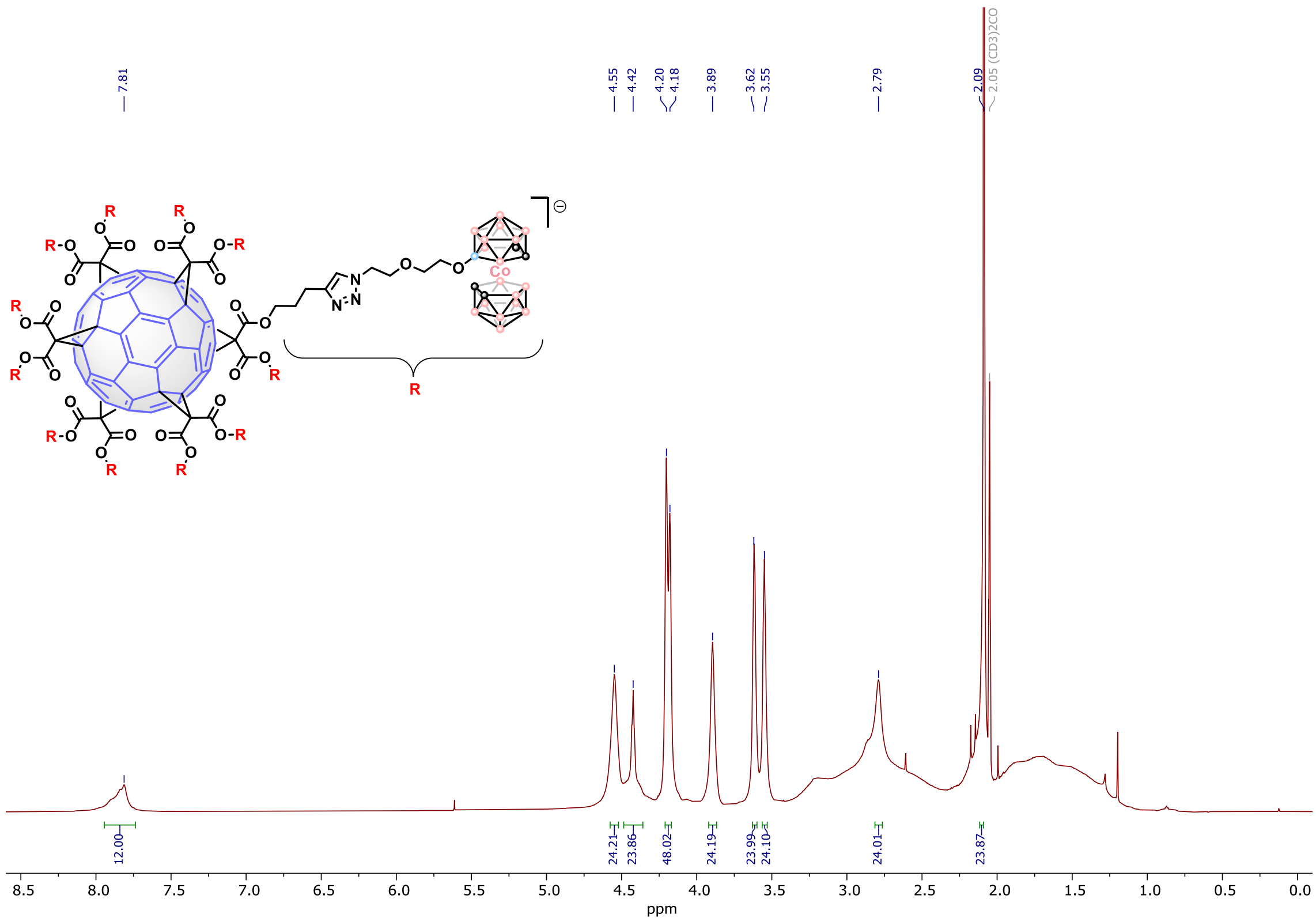

Figure S1. *^{1}H NMR spectrum of **C_{60}-COSAN** in acetone-d_6.*

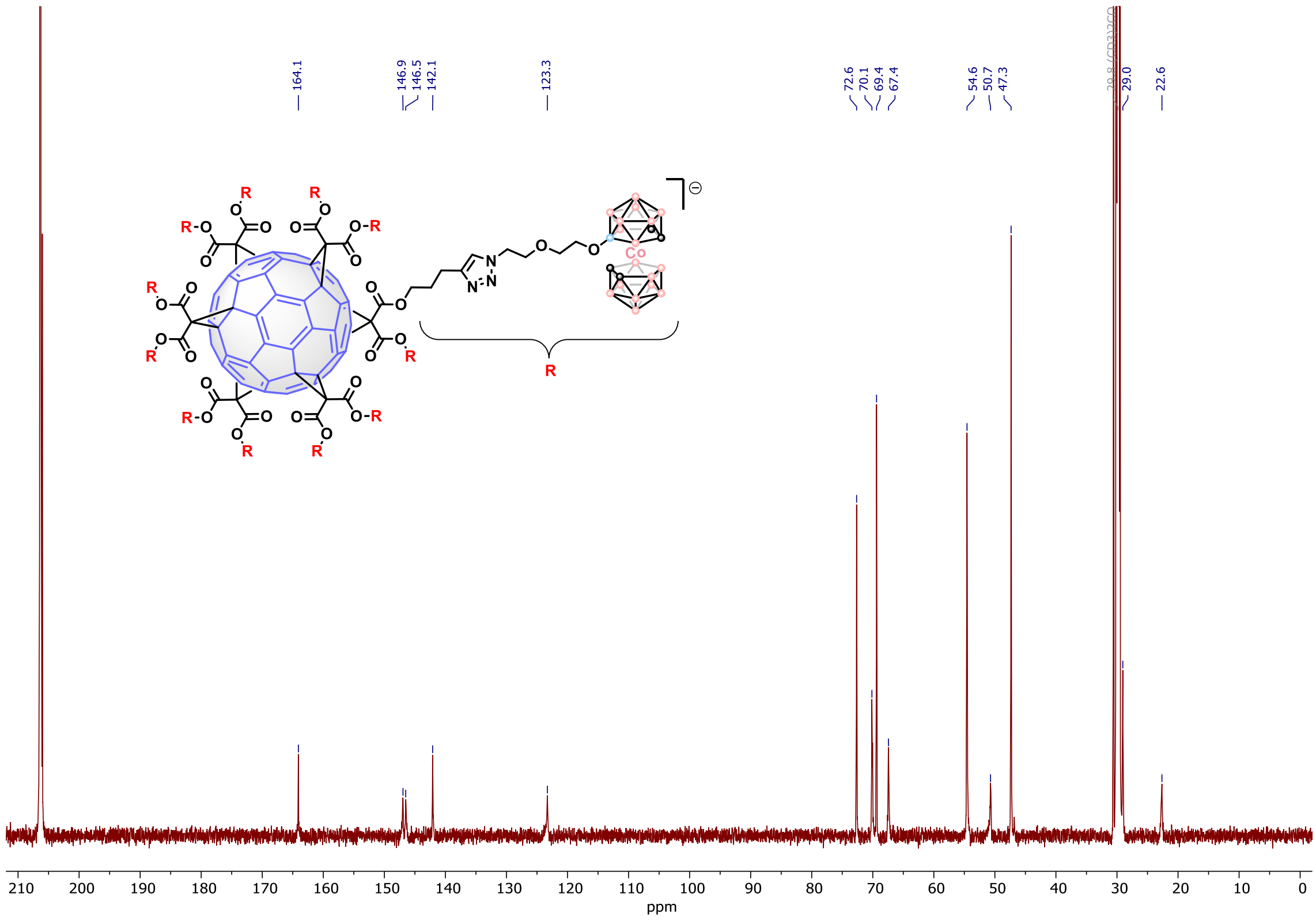

Figure S2. *^{13}C NMR spectrum of **C_{60}-COSAN** in acetone-d6.*

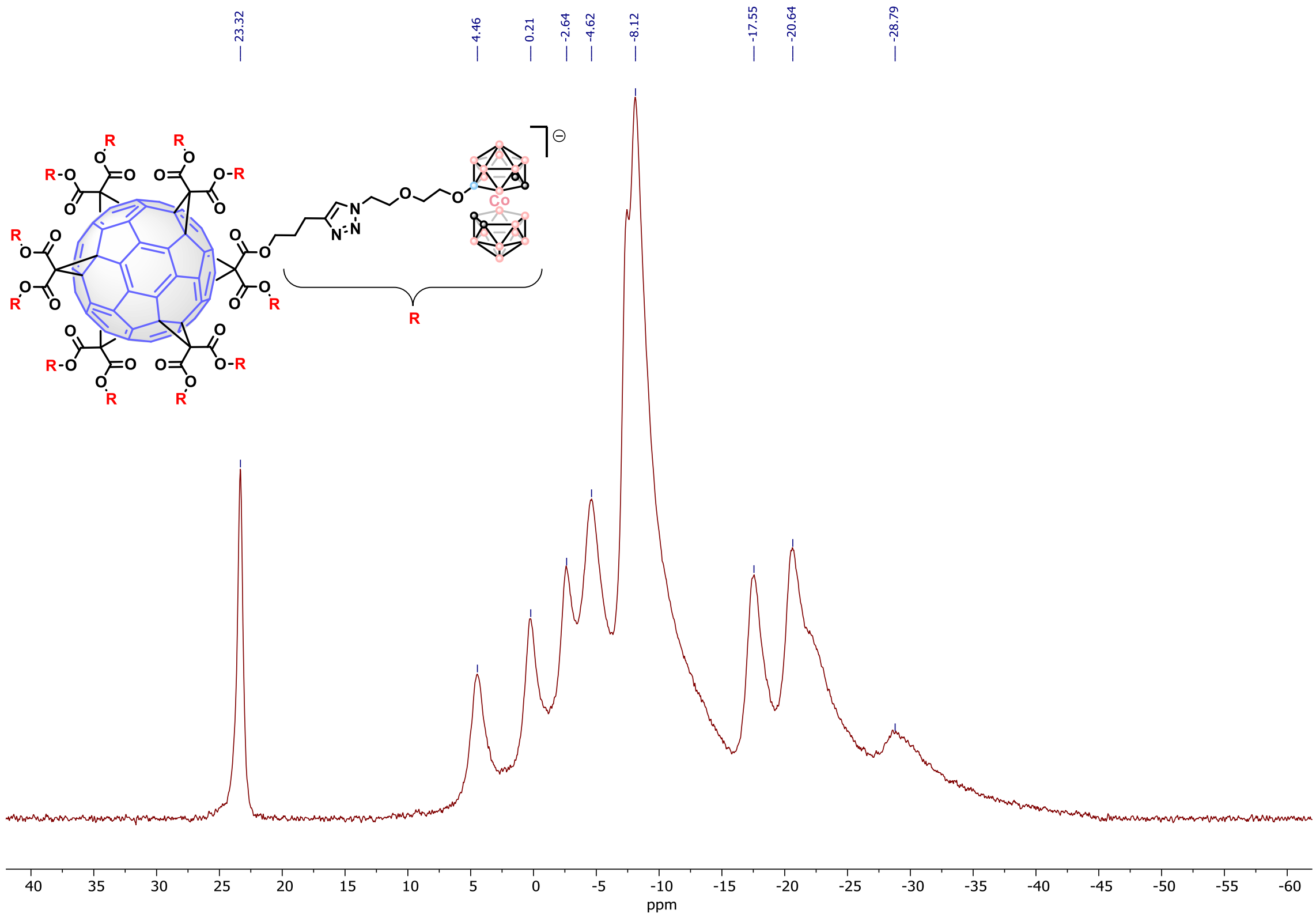

Figure S3. *^{11}B{^{1}H} NMR spectrum of **C_{60}-COSAN** in acetone-d6.*

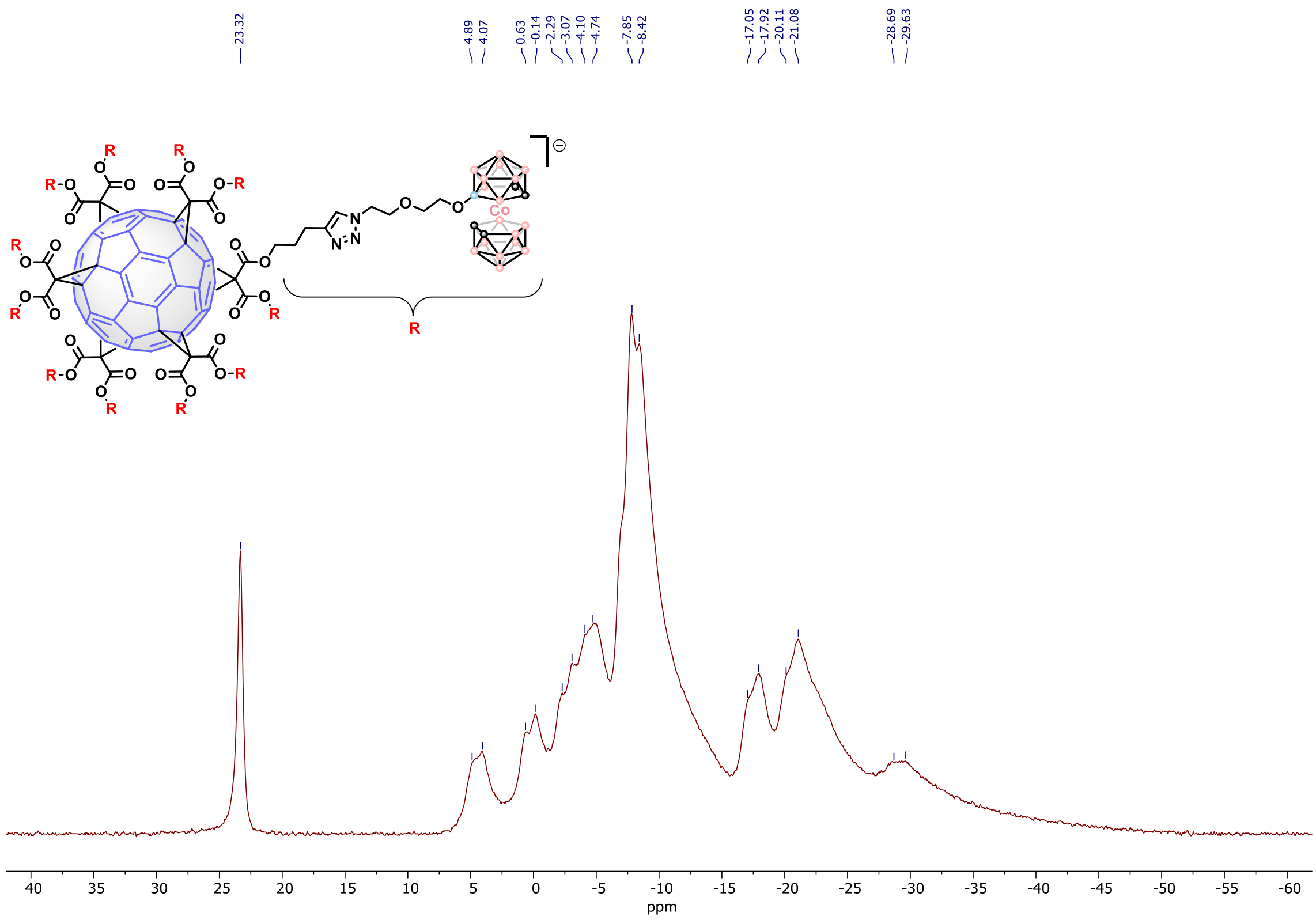

Figure S4. *^{11}B NMR spectrum of **C_{60}-COSAN** in acetone-d_6.*

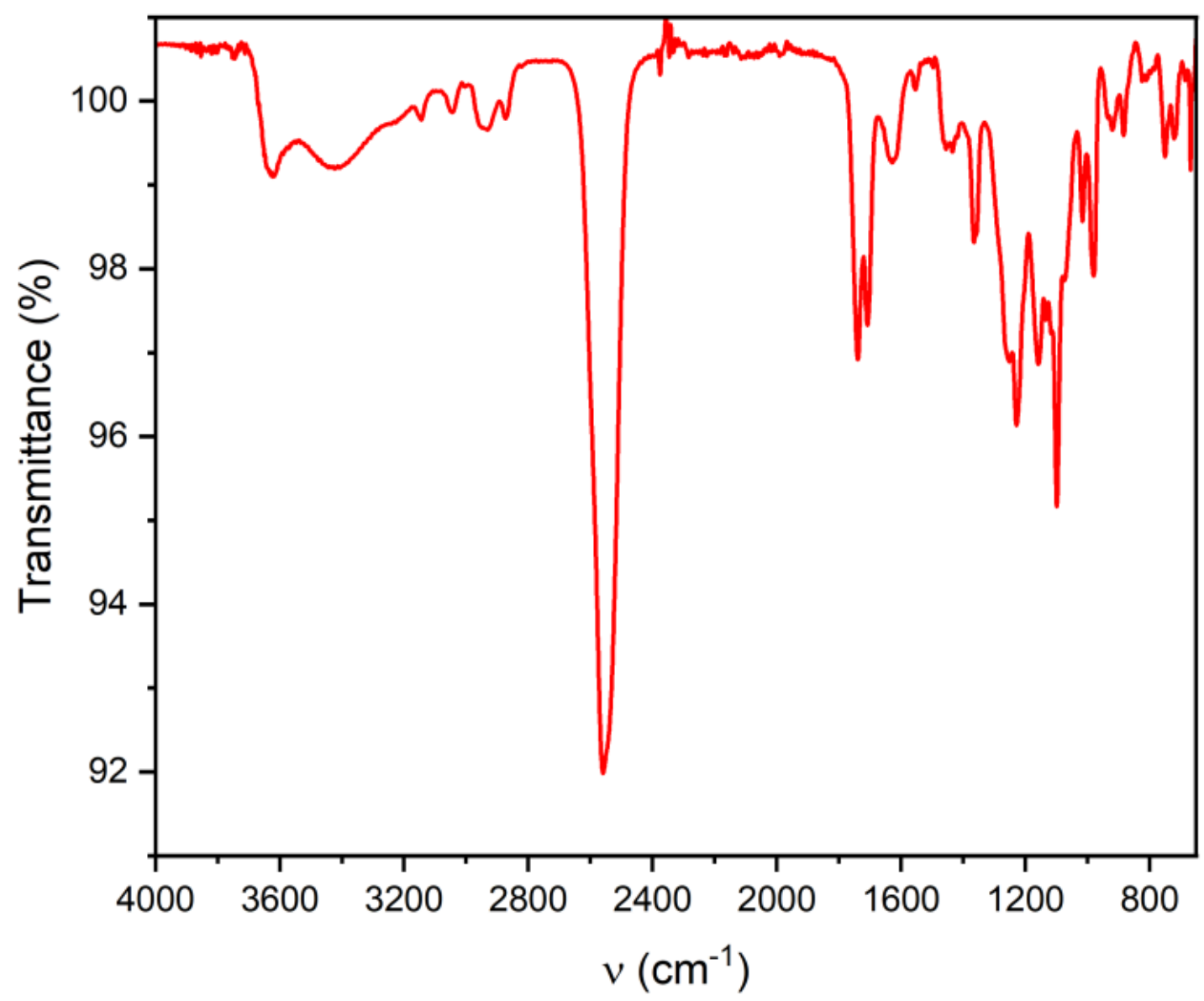

Figure S5. *FT-IR of **C_{60}-COSAN**.*

C_{60}-B_{12}

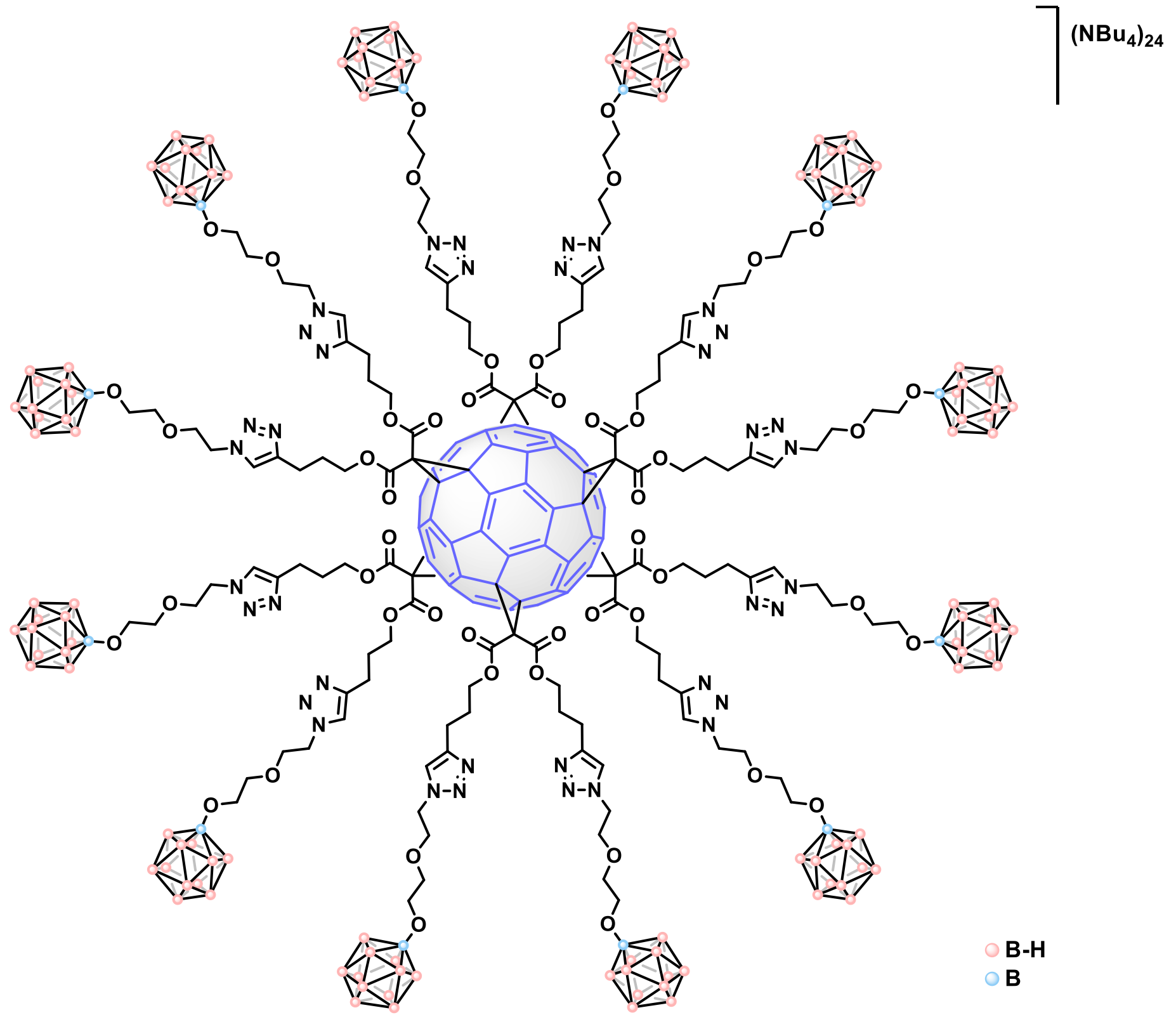

A mixture of compound **1** (20 mg, 9.6 µmol), compound **3** (113 mg, 0.15 mmol), $CuBr·S(CH_3)_2$ (16 mg, 0.097 mmol), sodium ascorbate (30 mg, 0.15 mmol) and a piece of copper metal wire in DMSO (1 mL) was deoxygenated and kept under argon with vigorous stirring for 72 h. The crude reaction was filtered through a Quadrasil® Mercaptopropyl column. Acetone was first added to the crude to precipitate compound **3** excess and then, to the filtrate, H_2O and $[NBu_4]Br$ sat. was added and filtered off to obtain **C_{60}-B_{12}** as an orange solid (65 mg, 60%). **^{1}H NMR** (700 MHz, acetone-d_6) δ: 8.18 (s, 12H; $CH_{triazole}$), 4.61 – 4.54 (m, 24H; OCH_2CH_2N), 4.54 – 4.37 (m, 24H; $COOCH_2$), 3.92 – 3.83 (m, 24H; CH_2O), 3.69 – 3.62 (m, 24H; CH_2O), 3.59 – 3.54 (m, 24H; CH_2O), 3.42 – 3.35 (m, 192; $NCH_2CH_2CH_2CH_3$), 2.86 – 2.73 (m, 24H; $COOCH_2CH_2CH_2$), 2.24 – 2.08 (m, 24H; $COOCH_2CH_2CH_2$), 1.81 – 1.74 (m, 192H; $NCH_2CH_2CH_2CH_3$), 1.44 (sx, $^3J_{H,H}$ = 7.4 Hz, 192H; $NCH_2CH_2CH_2CH_3$), 0.97 (t, $^3J_{H,H}$ = 7.5 Hz, 288H; $NCH_2CH_2CH_2CH_3$); **^{13}C NMR** (176 MHz, acetone-d_6) δ: 164.1 (CO), 146.6 ($C_{sp2\ fullerene}$), 146.5 ($C_{triazole}$), 142.1 ($C_{sp2\ fullerene}$), 124.4 ($CH_{triazole}$), 73.7, 70.1 (CH_2O), 69.1 (CH_2O and $C_{sp3\ fullerene}$), 68.0 ($COOCH_2$), 59.4 ($NCH_2CH_2CH_2CH_3$), 50.7 (OCH_2CH_2N), 29.2 ($COOCH_2CH_2CH_2$), 24.6 ($NCH_2CH_2CH_2CH_3$), 22.8 ($COOCH_2CH_2CH_2$), 20.4 ($NCH_2CH_2CH_2CH_3$), 14.1 ($NCH_2CH_2CH_2CH_3$); **^{11}B{^{1}H} NMR** (160 MHz, acetone-d_6) δ: 6.51 (s, 12B; BO), -16.51 (s, 60B; BH), -17.88 (s, 60B; BH), -22.64 (s, 12B; BH); **^{11}B NMR** (160 MHz, acetone-d_6) δ: 6.56 (s, 12B; BO), -14.52 – -20.48 (m, 120B; BH), -22.69 (d, $^1J_{B,H}$ = 96.8 Hz, 12B; BH); **FTIR-ATR** (ν cm^{-1}) = 2960 (m, CH), 2468 (s, BH), 1740 (w, CO).

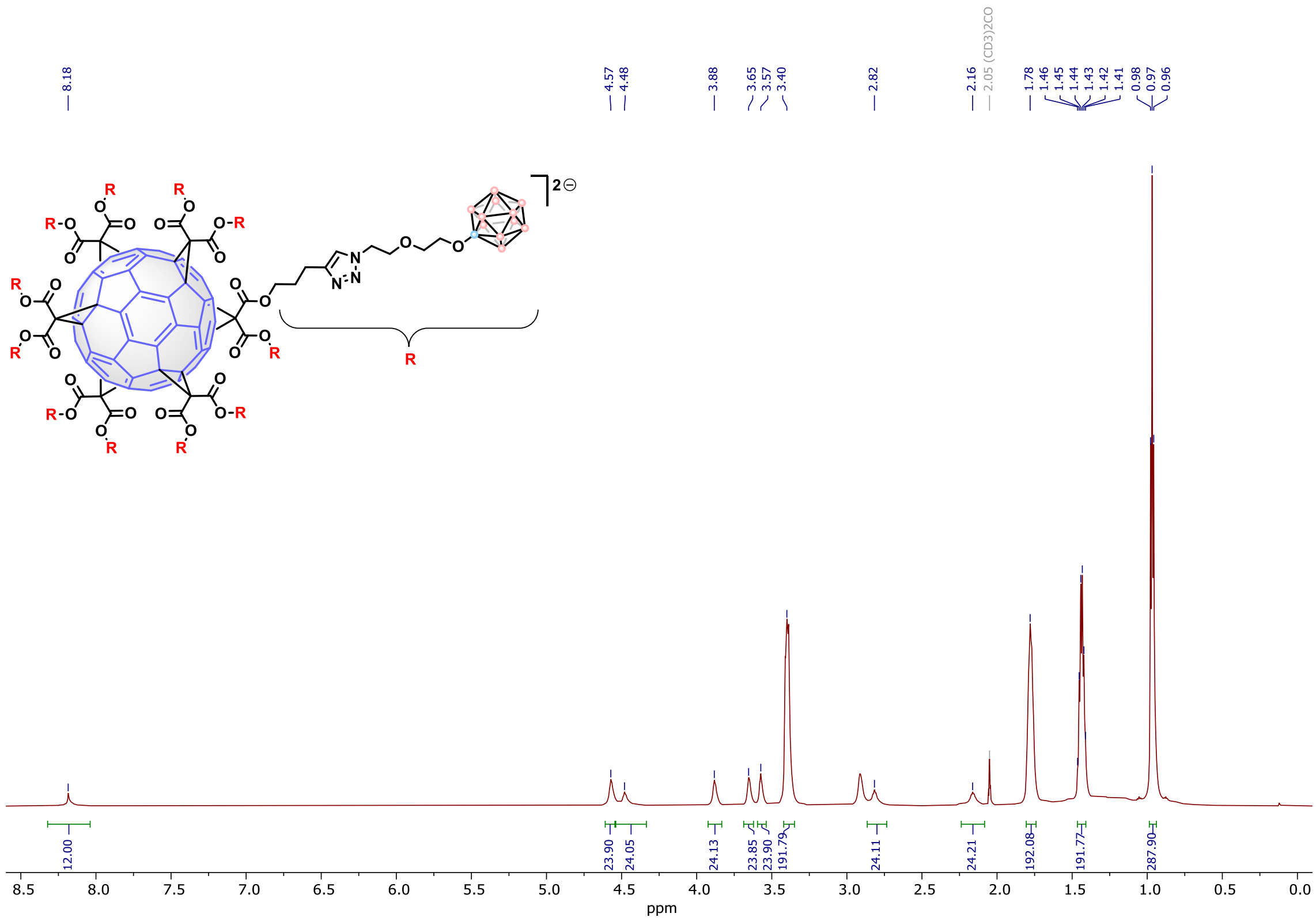

Figure S6. ^{1}H NMR spectrum of $\mathbf{C_{60}}$**-**$\mathbf{B_{12}}$ in acetone-d_6.

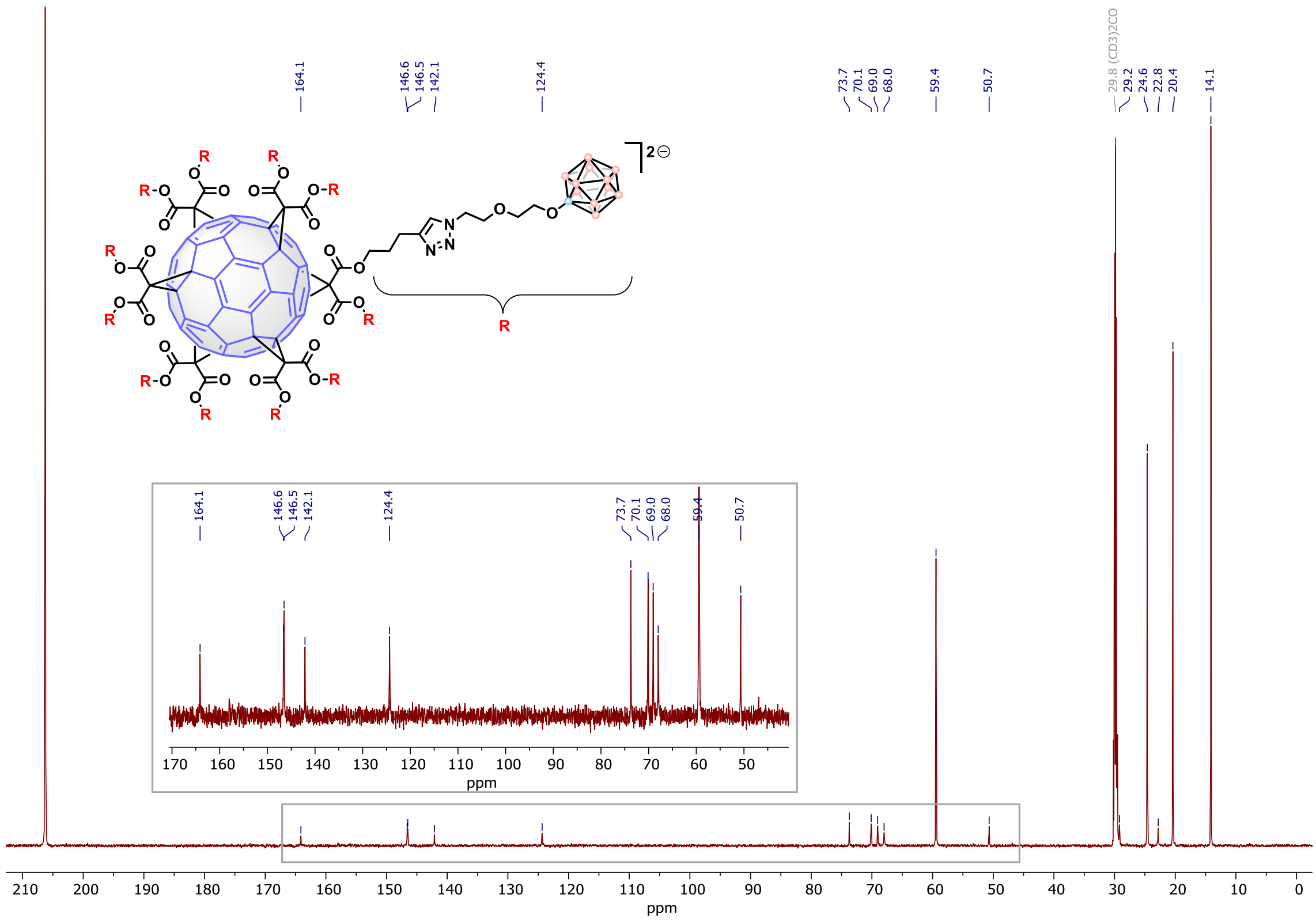

Figure S7. ^{13}C NMR spectrum of $\mathbf{C_{60}}$**-**$\mathbf{B_{12}}$ in acetone-d_6.

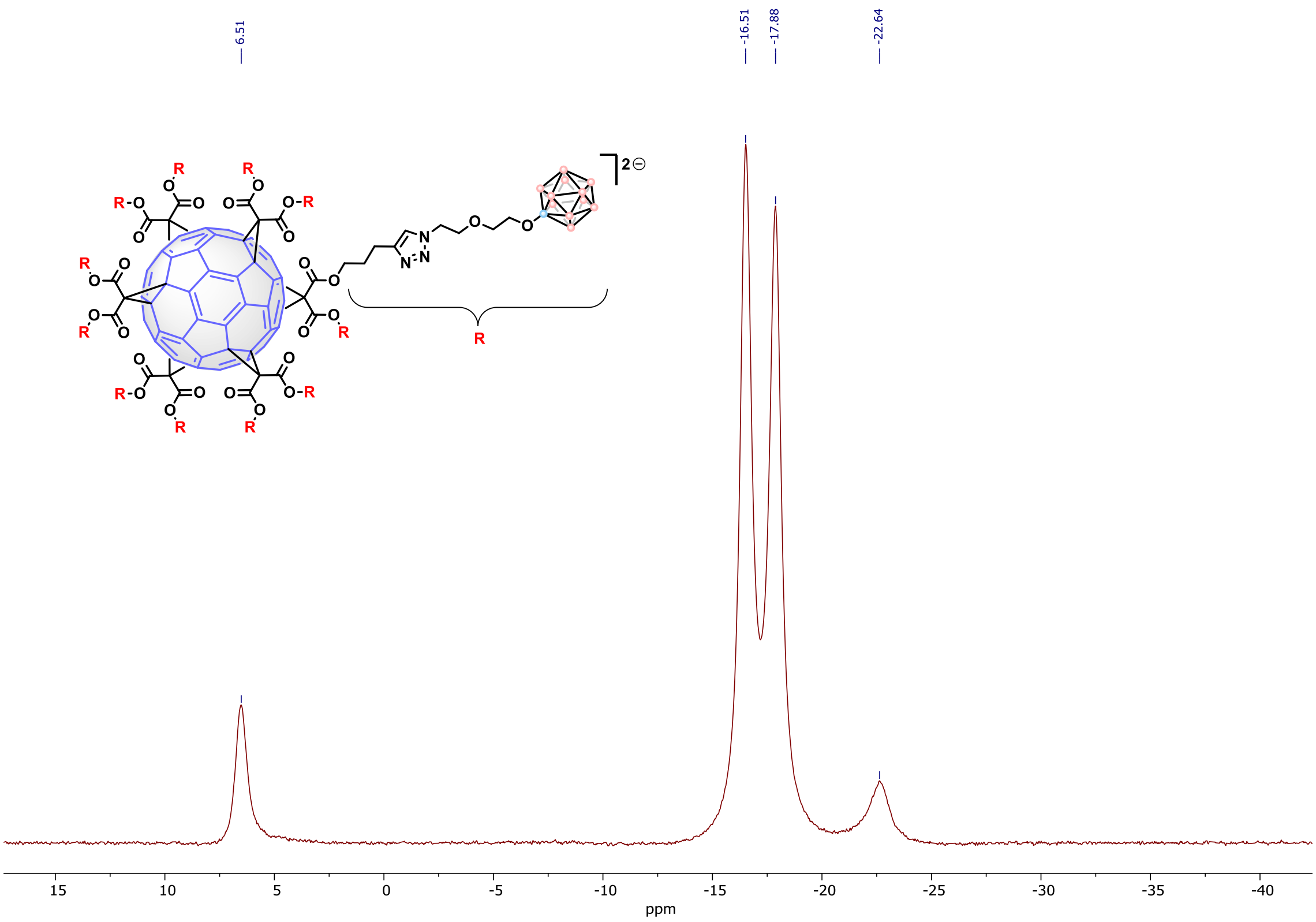

Figure S8. *^{11}B{^{1}H} NMR spectrum of* ***C_{60}-B_{12}*** *in acetone-d_6.*

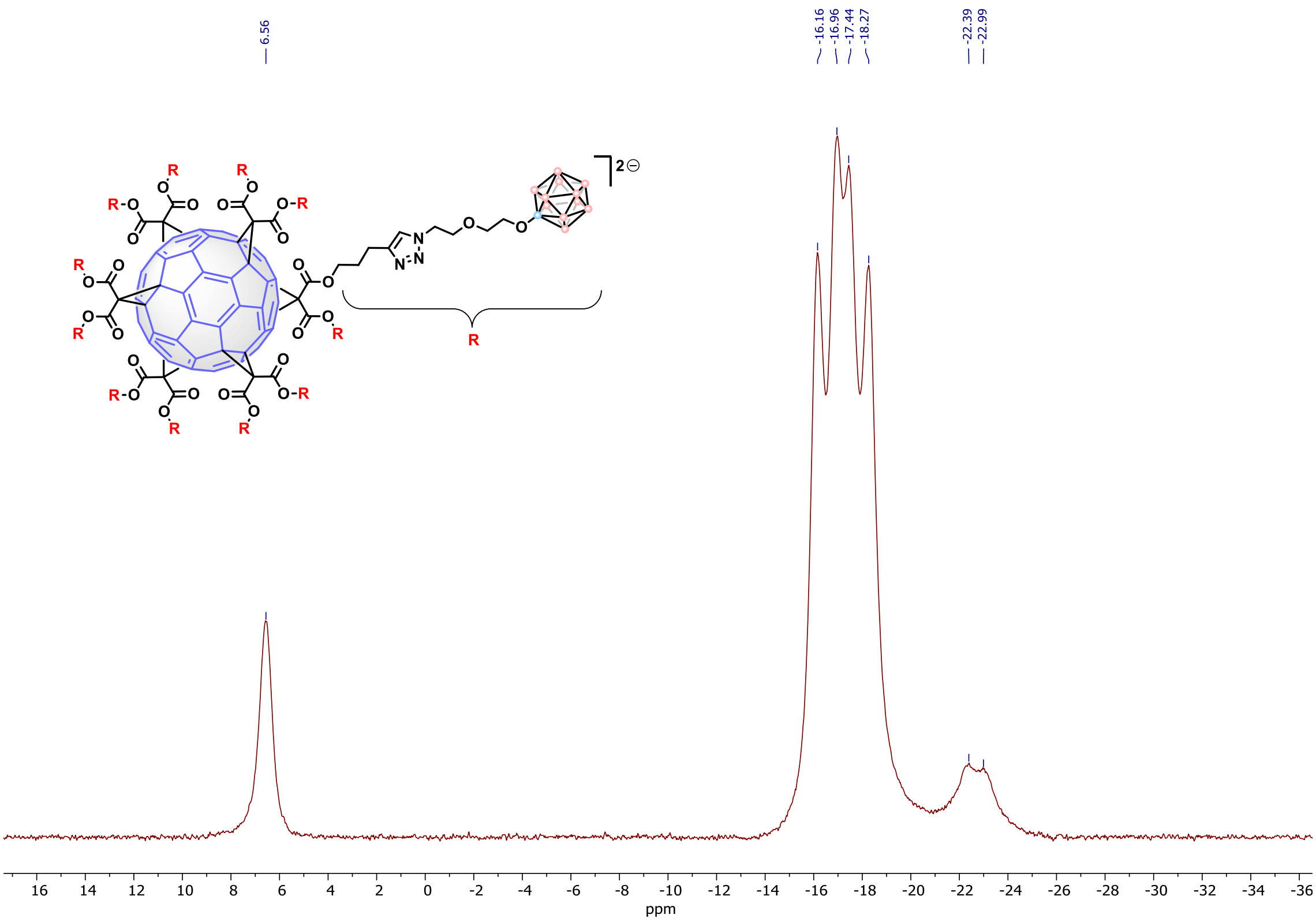

Figure S9. *^{11}B NMR spectrum of* ***C_{60}-B_{12}*** *in acetone-d_6.*

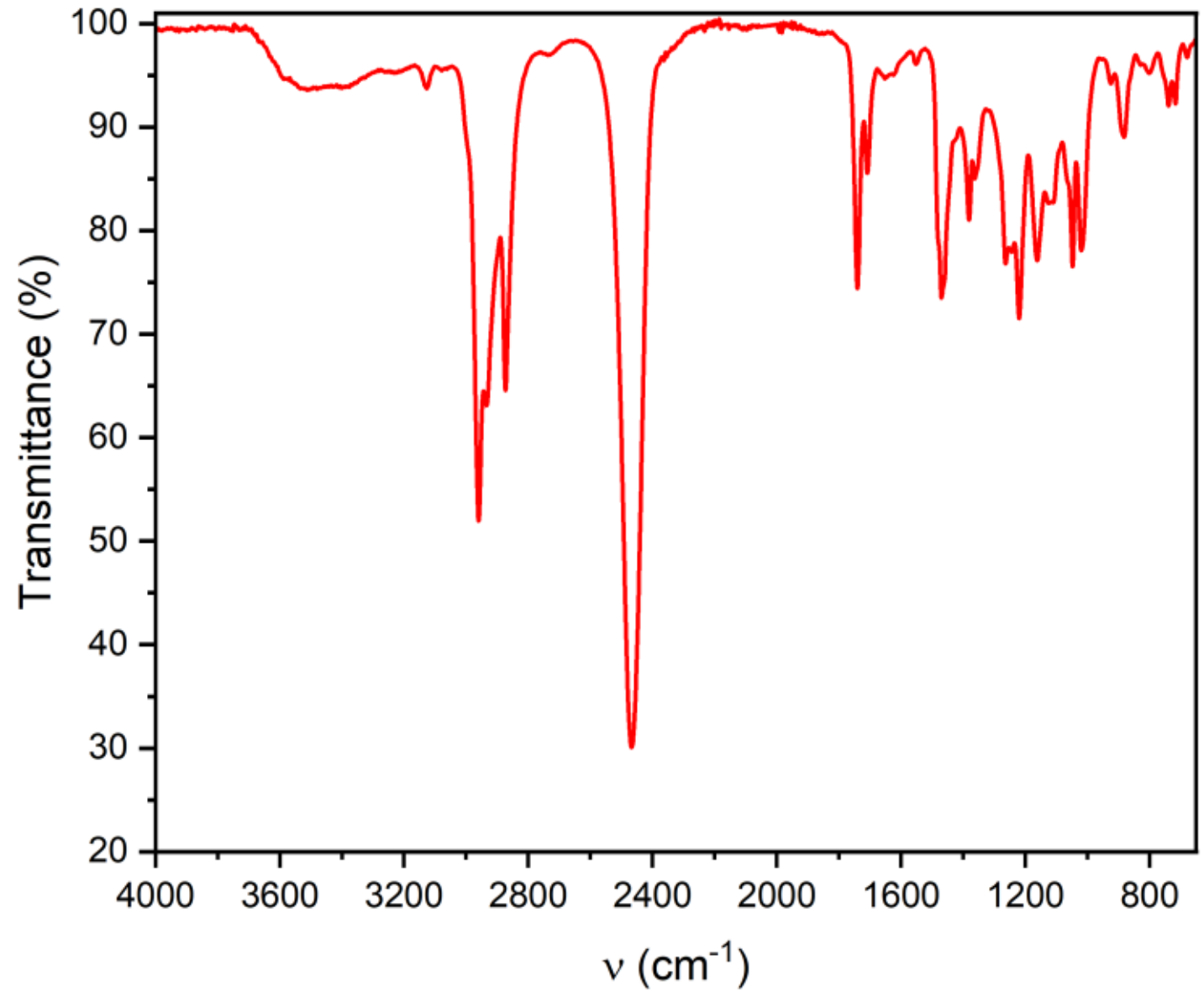

Figure S10. *FT-IR of* $\boldsymbol{C_{60}}$***-***$\boldsymbol{B_{12}}$.

2. Additional Characterization Data

2.1. UV-Vis spectra

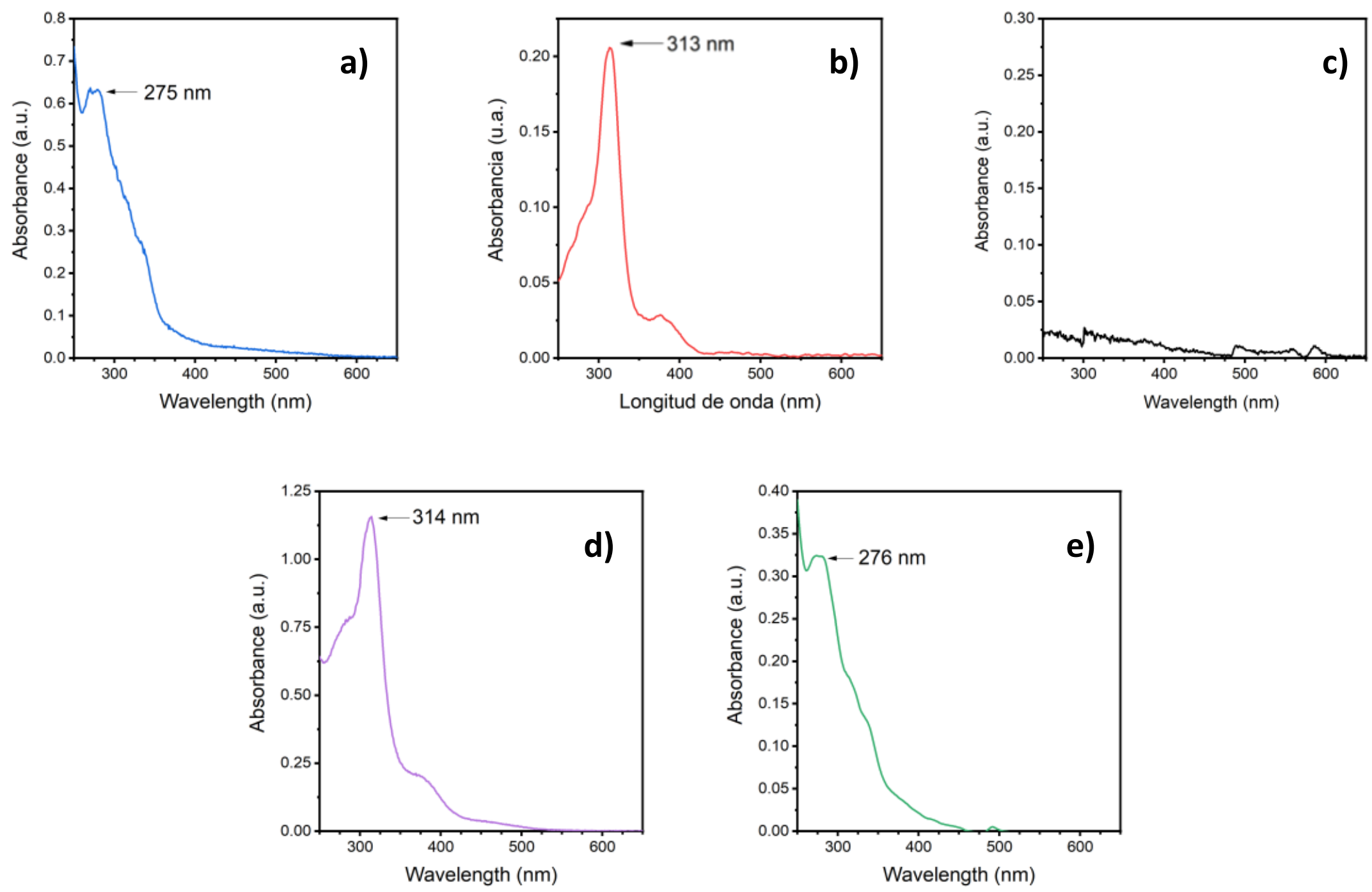

Figure S11. *UV-Vis spectra in* CH_3CN *of:* ***a)*** *Compound* ***1*** *1·10*$^{-6}$ *M,* ***b)*** *Compound* ***2*** *5·10*$^{-6}$ *M,* ***c)*** *Compound* ***3*** *1·10*$^{-5}$ *M,* ***d)*** $\boldsymbol{C_{60}}$***-COSAN*** *5·10*$^{-6}$ *M and* ***e)*** $\boldsymbol{C_{60}}$***-***$\boldsymbol{B_{12}}$ *5·10*$^{-6}$ *M.*

2.2. Cyclic voltametry

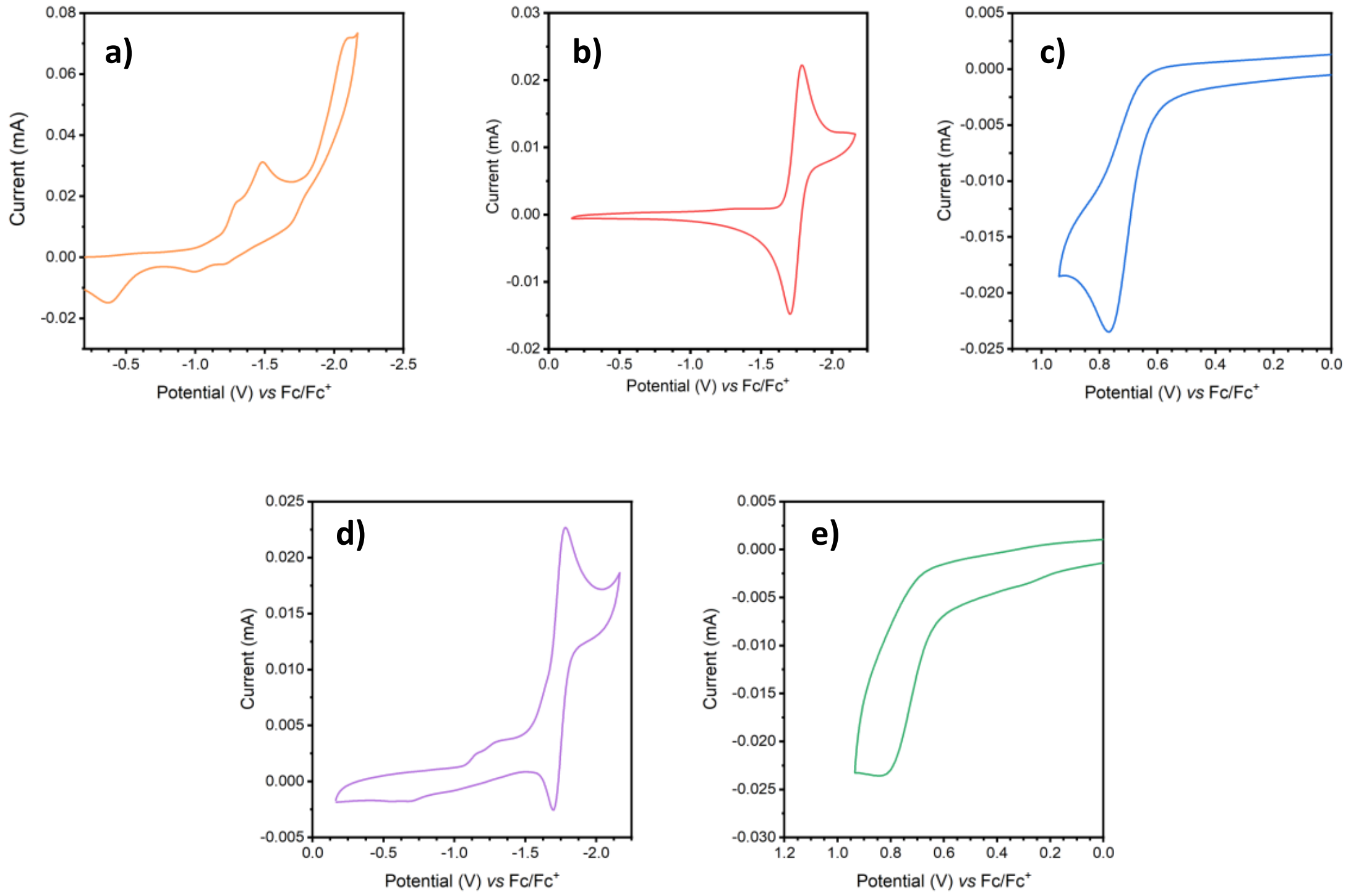

Figure S12. *Cyclic voltammograms in CH_3CN at 100 mV/s of:* ***a)*** *Compound* ***1*** *$1{\cdot}10^{-3}$ M,* ***b)*** *Compound* ***2*** *$1{\cdot}10^{-3}$ M,* ***c)*** *Compound* ***3*** *$1{\cdot}10^{-3}$ M,* ***d)*** ***C_{60}-COSAN*** *$1{\cdot}10^{-4}$ M and* ***e)*** ***C_{60}-B_{12}*** *$1{\cdot}10^{-4}$ M.*

2.3. Diffusion-Ordered NMR Spectroscopy (DOSY)

Table 1. Diffusion coefficients determined by 2D-DOSY (700 MHz, ~1 mg·mL^{-1} in acetone-d_6) and hydrodynamic radii.

Compound	Molecular weight (g/mol)	Diffusion coefficient (D, m^2·s^{-1})	Hydrodynamic radii (R_H, Å)
**C_{60}-COSAN**	7560.50	$3.52{\cdot}10^{-10}$	20.1
**C_{60}-B_{12}**	5377.45	$3.56{\cdot}10^{-10}$	19.9

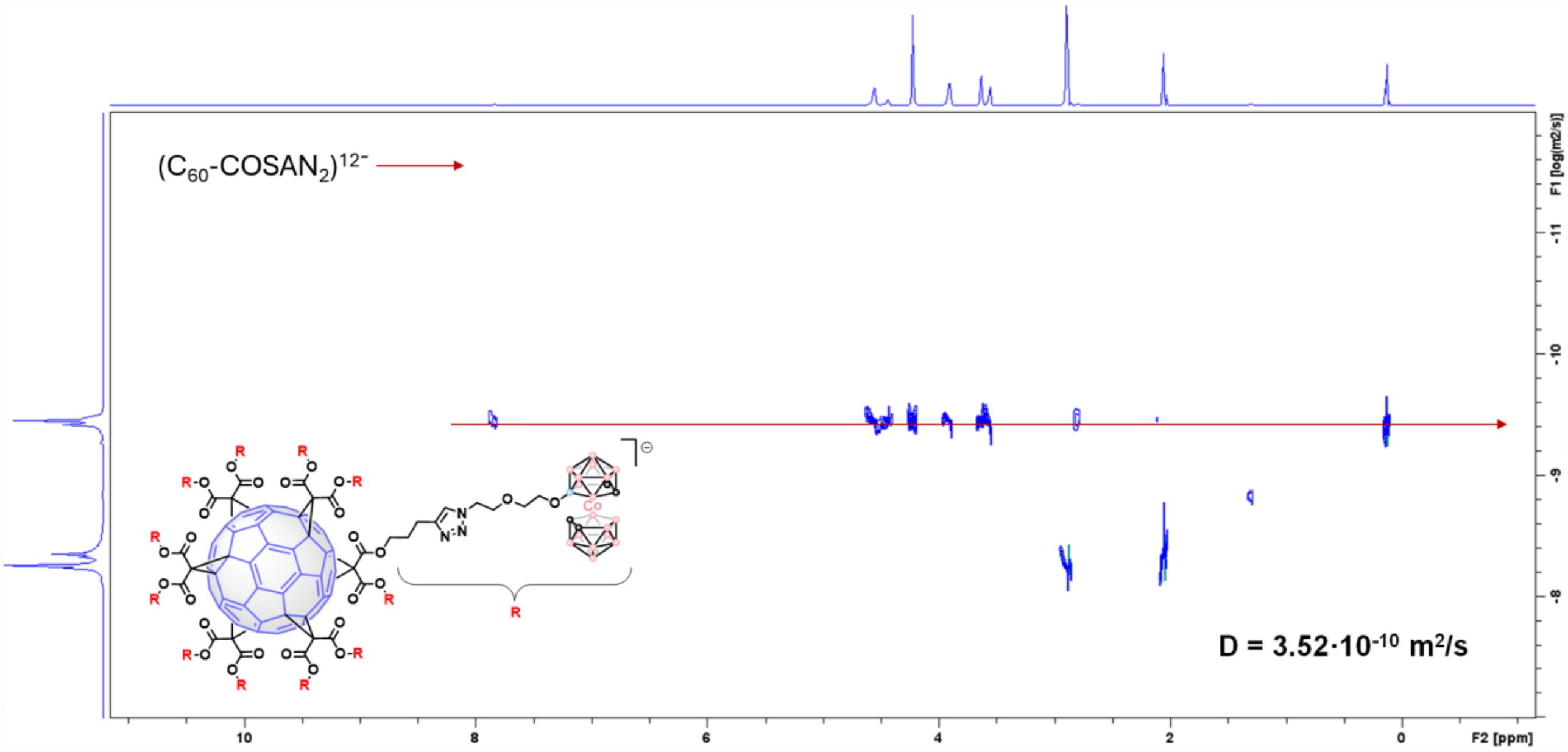

Figure S13. *DOSY-NMR spectrum of **C_{60}-COSAN** in acetone-d_6.*

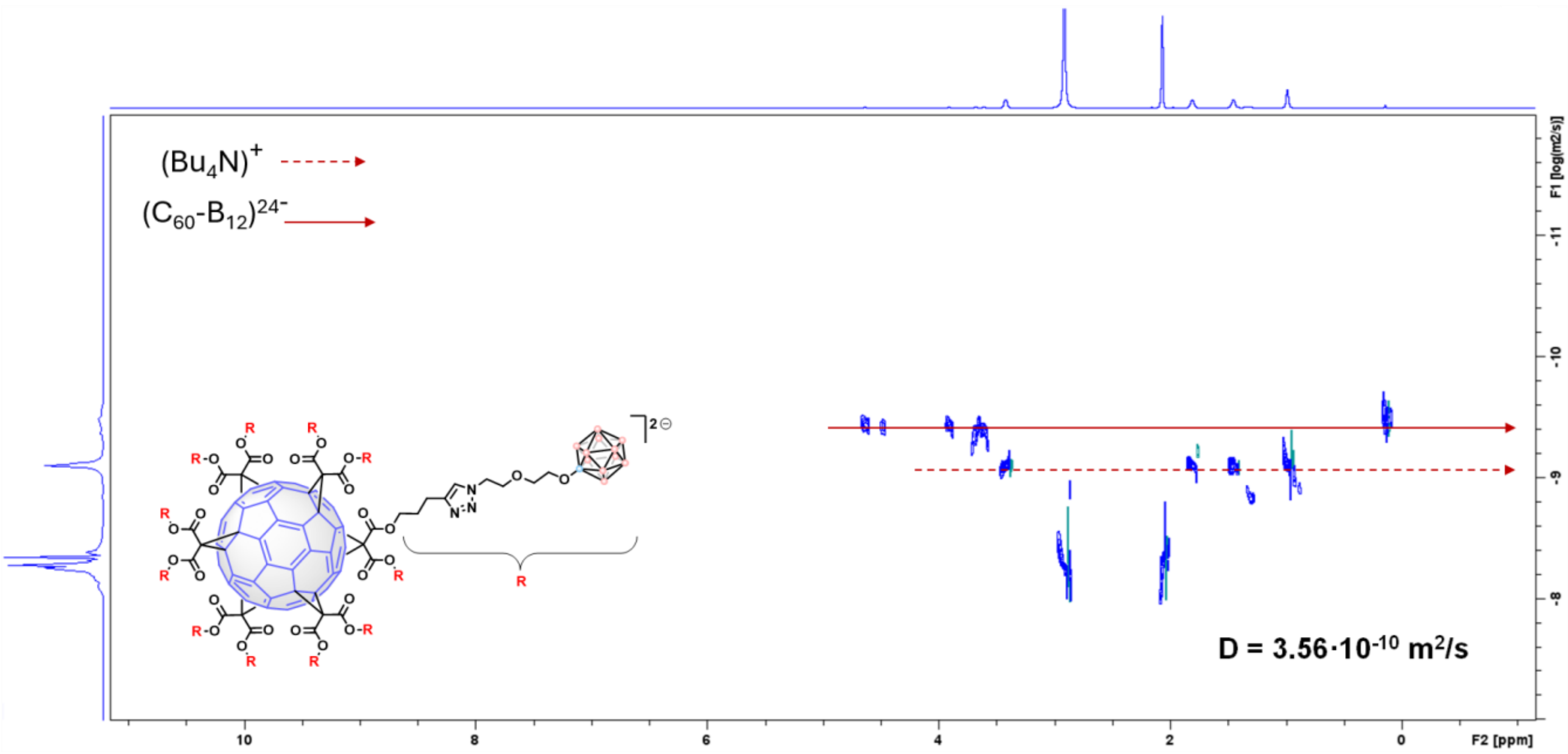

Figure S14. *DOSY-NMR spectrum of **C_{60}-B_{12}** in acetone-d_6.*

3. Biological Assays

*3.1 Preparation of liposome-encapsulated **C_{60}-COSAN** or **C_{60}-B_{12}***

1-palmitoyl-2-oleoyl-glycero-3-phosphocholine (POPC), cholesterol (Chol), 1,2-distearoyl-*sn*-glycero-3-phosphoethanolamine-*N*-[carboxy(polyethylene glycol)-2000] (sodium salt) (DSPE-PEG2000) and 1,2-dioleoyl-*sn*-glycero-3-phosphoethanolamine-*N*-(lissamine rhodamine B sulfonyl) (ammonium salt) (RhPE) were purchased from Avanti Polar Lipids (Merck). Liposomes composed of POPC/Chol/DSPE-PEG2000/RhPE at a molar ratio of 85/5/10/0.5 (mol/mol/mol/mol) (**Scheme**

S1) were prepared for the encapsulation of **C_{60}-COSAN** or **C_{60}-B_{12}** according to the protocol described below.

Appropriate volumes of lipid solutions in chloroform were transferred into glass tubes, followed by the addition of the required volumes of **C_{60}-COSAN** or **C_{60}-B_{12}** dissolved in absolute ethanol. The mixtures were vortexed to ensure homogeneous mixing of all components, after which the organic solvents were removed under reduced pressure using a SpeedVac concentrator for 30 min, yielding a thin lipid film containing the C_{60}-based compounds. The lipid film was subsequently hydrated with 500 µL of 100 mM HEPES buffer (pH 7.4), resulting in final lipid concentrations of 3 mg/mL and 2 mg/mL for the preparation of liposome-encapsulated **C_{60}-COSAN** and **C_{60}-B_{12}**, respectively. Hydration was facilitated by multiple cycles of vortexing and sonication in a water bath. The resulting multilamellar vesicle suspensions were reduced in size by 1–2 cycles of probe sonication (10 min per cycle) using a tip sonicator, yielding nanosized liposomal formulations.

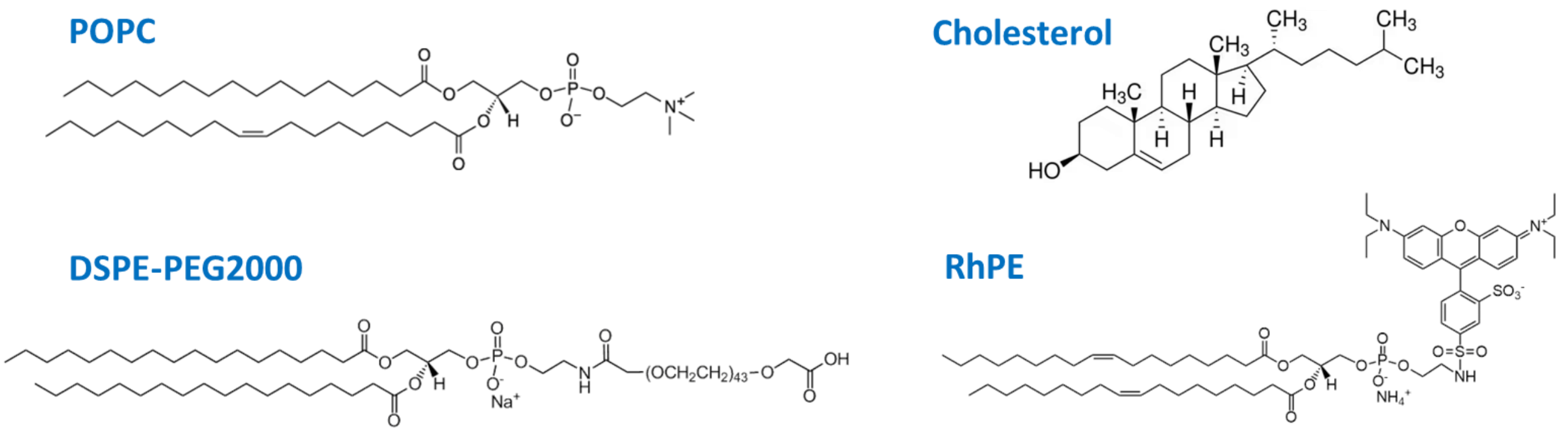

Scheme S1. *Structure of the different lipids used to form liposomes.*

3.2. Particle size, polydispersity and ζ-Potential

The average hydrodynamic diameter (Z-average) and polydispersity index (PDI) were determined by dynamic light scattering (DLS) using a Zetasizer (Malvern Instruments) equipped with a 633 nm He–Ne laser. Scattered light was detected at a backscattering angle of 173° using homodyne detection. Measurements were performed in high-performance quartz cuvettes (10 mm path length, 3,500 µL chamber volume, spectral range 200–2,500 nm) at 298 K without sample dilution. For each sample, three technical replicates were acquired, and the reported values correspond to the mean ± standard deviation. Particle size results are reported as the intensity-weighted Z-average values calculated using the cumulants method.

The ζ-potential was measured using the same instrument with scattered light detected at 13°. Measurements were performed in DTS1070 folded capillary cells (Malvern Instruments) by applying

a voltage of 20 mV between the electrodes. Three technical replicates were recorded for each sample, and the results are presented as mean ± standard deviation.

3.3. Encapsulation efficiency, drug loading capacity and cargo retetion

The encapsulation efficiency (EE) and drug loading (DL) of the boron-loaded liposomal formulations were determined by UV–Vis spectroscopy using a GENESYS™ 10S UV–Vis spectrophotometer (Thermo Scientific, Waltham, MA, USA). Absorption spectra of the liposomal suspensions were first recorded between 250 and 400 nm with a spectral resolution of 1 nm. The dispersions were then ultracentrifuged at 436,000 × g (100,000 rpm, TLA-100.3 rotor, Beckman Coulter) for 20 min at 4 °C to separate the non-encapsulated boron hybrids from the liposomal fraction. The supernatants were carefully collected, and their absorption spectra were recorded under identical conditions. The concentrations of the boron hybrids were determined using the absorbance at 276 nm for **C_{60}-COSAN** and 314 nm for **C_{60}-B_{12},** corresponding to the absorption maxima of each compound (**Figure S11**). At these wavelengths, the absorbance of the boron hybrids was sufficiently high to minimize the influence of light scattering from the liposomal suspensions, enabling reliable quantification of the non-encapsulated fraction.

The encapsulation efficiency (EE) was calculated from the amount of non-encapsulated compound remaining in the supernatant according to:

$$EE(\%) = \left(1 - \frac{A_{\text{supernatant}}}{A_{\text{initial}}}\right) \times 100$$

where A_{initial} is the absorbance corresponding to the total amount of boron hybrid initially added during liposome preparation and $A_{\text{supernatant}}$ is the absorbance of the non-encapsulated hybrid measured in the supernatant after ultracentrifugation.

Drug loading (DL) was calculated according to:

$$DL(\%) = \frac{m_{\text{encapsulated}}}{m_{\text{lipids}}} \times 100$$

where $m_{\text{encapsulated}}$ corresponds to the amount of encapsulated boron hybrid, calculated as the difference between the initial amount of hybrid and the amount quantified in the supernatant, and m_{lipids} is the total mass of lipids used for liposome preparation.

For stability studies, the apparent encapsulation efficiency after 72 h was determined following the same procedure. Cargo retention was subsequently calculated according to:

$$\text{Cargo retention}(\%) = \frac{EE_{72}}{EE_0} \times 100$$

where $EE_0$and EE_{72}correspond to the encapsulation efficiencies measured immediately after preparation and after 72 h of incubation, respectively.

3.4. Cell culture

Mouse embryonic fibroblasts (MEFs) were purchased from ATCC (CRL-2991). Cells were cultured in complete Dulbecco Modified Eagle Medium (DMEM) supplemented with 10% Premium Fetal bovine serum (FBS, South Africa S1300; Biowest, Nuallé, France), 1% non-essential amino acids (MEM-NEA), 1% penicillin/ Streptomycin (final concentration of 100 U/mL of penicillin and 100 µg/mL of streptomycin), all purchased from Gibco (Carlsbad, CA, USA). Cells were grown in a humidified incubator (Forma Steri-Cycle ThermoFisher with HEPA fil ter, 5% CO_2) at 37 ºC and maintained with a split ratio of 1:15 at 80% of confluence in T75 flasks (Nunc). Trypsine/ EDTA 0.05% from Hyclone was used to detach cell cultures from flasks. Dulbecco Phosphate Buffered Saline without calcium and magnesium (DPBS) from Gibco and Phosphate Buffered Saline 1x (PBS) from Hyclone, were used for washing cells.

The head and neck squamous cell carcinoma cell line Cal33 was kindly provided by the Institute of Advanced Biosciences, Grenoble. Cells were maintained in DMEM medium supplemented with 10% FBS and 1% penicillin/streptomycin (all from Gibco, at 80% confluence in a humidified atmosphere with 5% CO_2 at 37 °C.

3.5. Confocal laser scanning microscopy

Cellular uptake and localization of liposome-encapsulated **C_{60}-COSAN** or **C_{60}-B_{12}** were investigated in Mouse Embryonic Fibroblasts (MEFs) and Cal33 cells. Liposomal formulations were administered at final lipid concentrations of 0.3 mg/mL for **C_{60}-COSAN** and 0.2 mg/mL for **C_{60}-B_{12}** in the well, and at a final boron concentration of 10 µg/mL. Experiments were performed using an inverted Nikon Ti-E microscope equipped with a Nikon C2 confocal scanning module, a Nikon Plan Apo 100× oil immersion objective (NA 1.45), and two excitation lasers (488 and 561 nm; Sapphire laser). Cells were seeded at a density of 1 × 10^4 cells/cm^2 in 8-well Nunc™ Lab-Tek™ chamber slides (Thermo Fisher Scientific) containing 250 µL of complete DMEM per well and treated with liposome-encapsulated **C_{60}-COSAN** or **C_{60}-B_{12}**. To assess subcellular localization of the nanovectors, cells

were pre-incubated with LysoTracker Green (1 µM), a fluorescent probe specific for lysosomes, prior to treatment with the liposomal formulations. Cells were imaged at different time points (0, 2, 24, and 48h). Colocalization between the liposome-associated Rhodamine-PE signal (red channel) and the LysoTracker Green signal (green channel) was evaluated by overlaying the corresponding fluorescence channels. Signal overlap was taken as indicative of lysosomal localization of the internalized liposomes, consistent with uptake via endocytic pathways. Confocal images were analyzed using ImageJ software.

3.6. Cell viability assays

Cell viability was assessed using the alamarBlue assay (Thermo Fisher Scientific) according to the manufacturer's instructions. Cells were seeded at a density of 1,000 cells per well in 96-well plates containing 100 µL of complete growth medium and cultured until approximately 80% confluence at 37 °C under a humidified atmosphere with 5% CO_2. After 24h, cells were washed with DPBS and re-incubated in complete growth medium containing liposome-encapsulated **C_{60}-COSAN** or **C_{60}-B_{12}** at different boron concentrations (2.1, 5.2, 10, 15.63, and 26.04 µg/mL) for 24 and 48h under the same culture conditions.

AlamarBlue reagent (10% v/v) was added to each well 2h prior to absorbance measurement using a Multiskan FC plate reader (Thermo Fisher Scientific). Absorbance was measured at 570 nm, with 620 nm used as the reference wavelength. Cell viability was evaluated in three technical replicates. Values were normalized to the untreated control (100% viability, dashed line in **Figures 4a** and **5a**), and the observed differences represent technical variability within the assay. No statistical comparisons between conditions were performed because no independent biological replicates were available.

BIBLIOGRAPHY

[1] M. Sánchez-Navarro, A. Muñoz, B. M. Illescas, J. Rojo, N. Martín, *Chem. Eur. J.* **2011**, *17*, 766-769.
[2] B. A. Wojtczak, A. Andrysiak, B. Grüner, Z. J. Lesnikowski, *Chem. Eur. J.* **2008**, *14*, 10675-10682.
[3] A. V. Orlova, N. N. Kondakov, B. G. Kimel, L. O. Kononov, E. G. Kononova, I. B. Sivaev, V. I. Bregadze, *Appl. Organometal. Chem.* **2007**, *21*, 98-100.
